# Investigating the Antimycobacterial, Antibiofilm, and Antioxidant Activities of Plant Extracts Against *Mycobacterium smegmatis*

**DOI:** 10.3390/microorganisms14051133

**Published:** 2026-05-16

**Authors:** Ramokone Mothupi, Mashilo Matotoka, Gabriel Mashabela, Peter Masoko

**Affiliations:** 1 Department of Biochemistry, Microbiology and Biotechnology, Faculty of Science and Agriculture, University of Limpopo, Private Bag X1106, Sovenga 0727, South Africa; ramokoneflora@gmail.com (R.M.); mashilo.matotoka@ul.ac.za (M.M.); 2 Division of Molecular Biology and Human Genetics, SAMRC Centre for Tuberculosis Research, Stellenbosch University, Tygerberg Campus, Francie Van Zijl Drive, Parow, Cape Town 7505, South Africa; gabrielm@sun.ac.za

**Keywords:** *Mycobacterium tuberculosis*, *Mycobacterium smegmatis*, plant extracts, antimycobacterial activity, antibiofilm, anti-sliding motility

## Abstract

The persistence of *Mycobacterium tuberculosis* within biofilm-like structures underscores the need for alternative drug discovery strategies aimed at resistance mechanisms. Medicinal plants provide a rich source of chemically diverse compounds with broad biological activities, including potential antimycobacterial properties. This study investigated acetone stem extracts from *Buddleja saligna*, *Combretum hereroense*, and *Olea europaea* subsp. *africana* for their phytochemical composition, antioxidant capacity, and antimycobacterial activity against planktonic and biofilm forms of *Mycobacterium smegmatis*. Phytochemical profiles were analyzed using liquid chromatography–mass spectrometry and quantified through colorimetric assays. Antioxidant activity was assessed using DPPH radical scavenging and ferric reducing power assays, while antimycobacterial effects at MIC and sub-MIC levels were determined through microdilution and growth kinetic assays. Phytochemical composition and concentrations varied among extracts, with *B. saligna* exhibiting the highest levels of tannins (287.18 ± 0.19 mgGAE/g extract) and flavonoids (16.48 ± 0.05 mgQE/g extract) and showing the strongest antioxidant activity (17.66 ± 5.396 and 399.1 ± 3.717 µg/mL). *C. hereroense* had a notable antimycobacterial activity with an MIC of 0.16 mg/mL followed by *B. saligna* and *O. europaea* subsp. *afriana* with MIC values of 0.31 and 0.63 mg/mL, respectively. All extracts significantly inhibited early biofilm formation by over 80% at sub-MICs. However, the mature biofilms and sliding motility were less susceptible to the extracts. Overall, the results confirm the antioxidant and antimycobacterial potential of the selected plant extracts, while highlighting challenges in targeting mycobacterial biofilms.

## 1. Introduction

Tuberculosis (TB), caused by Mycobacterium tuberculosis, remains a major global public health concern. Its burden is exacerbated by the emergence of multidrug-resistant (MDR) strains, which are resistant to first-line antitubercular drugs such as rifampicin and isoniazid, extensively drug-resistant (XDR) strains which show resistance to both first-line and second-line antibiotics, as well as the adverse effects associated with conventional treatment regimens. TB continues to rank among the leading causes of mortality from infectious diseases worldwide [1,2,3]. The pathogen is transmitted via airborne droplets and primarily targets the respiratory system, posing increased risk to immunocompromised individuals and those with pre-existing lung conditions [4,5]. According to the World Health Organization 2025 Global Tuberculosis Report, an estimated 10.7 million cases and 1.23 million deaths occurred in 2024, although incidence and mortality declined for the first time since 2020. Despite this progress, timely diagnosis and effective treatment remain inaccessible to many due to ongoing financial and healthcare system constraints [6,7].

*Mycobacterium tuberculosis* employs multiple adaptive strategies that enhance its persistence and pathogenicity, notably biofilm formation and sliding motility. Biofilms are structured bacterial communities embedded within an exopolysaccharide-rich matrix that restricts antibiotic penetration, thereby promoting drug tolerance and contributing to persistent and recurrent tuberculosis infections [8,9]. The expansion and surface colonization of mycobacterial biofilms are facilitated by sliding motility, a passive form of translocation mediated by surface-active molecules such as exopolysaccharides, glycopeptidolipids (GPLs), and saturated polyesters [10]. Evidence from *Mycobacterium smegmatis* indicates that GPL-dependent motility enhances rapid surface spreading and confers a competitive advantage, while disruption of motility impairs biofilm development, highlighting a functional interdependence between these processes. Collectively, these adaptations underscore the critical interplay between sliding motility and biofilm formation in mycobacterial survival and identify a potential therapeutic target for controlling biofilm-associated infections [11,12]. Consequently, biofilm-associated bacteria exhibit significantly greater resistance to antimicrobial agents than their planktonic counterparts, contributing to the persistence and recurrence of chronic infections, including tuberculosis [13,14]. In addition, initial tuberculosis infection triggers a cascade of host immune responses, including macrophage activation and the production of reactive oxygen species (ROS) aimed at pathogen clearance. However, *M. tuberculosis* has evolved sophisticated immune evasion mechanisms that enable intracellular survival despite oxidative stress. While ROS are central to antimicrobial defense, their excessive accumulation can induce oxidative stress, leading to host tissue damage, inflammation, and increased disease severity [15,16]. This dynamic interplay between host immune pressure and bacterial resilience further emphasizes the need for novel therapeutic strategies and a deeper mechanistic understanding of *M. tuberculosis* pathogenesis.

Natural products, particularly medicinal plants, have long been recognized as valuable sources for treating infectious diseases such as TB. Previous studies have demonstrated their therapeutic potential, especially in targeting bacterial behaviors such as biofilm formation and motility [17]. Some of the natural products with antitubercular and antibiofilm potency include *Artemisia afra* [18], *Salvia aurea* and *Sphedamnocarpus pruriens* [19]. The effectiveness of these natural remedies is largely attributed to their diverse phytochemical constituents, which exhibit a broad spectrum of biological activities, including antimicrobial, anti-inflammatory, and antioxidant effects, among others. These properties make medicinal plants promising candidates for the development of novel anti-TB therapies that can complement or enhance existing treatment strategies [20,21]. The plants selected for this study (*Buddleja saligna* (Willd.), *Combretum hereroense* Schinz (russet bushwillow), and *Olea europaea* subsp. *africana* (Mill.)) were chosen based on their traditional use for treating TB and related symptoms [22,23,24,25,26,27,28]. *Buddleja saligna* (Willd.), a member of the Scrophulariceae family, is an evergreen tree of small to medium size that is indigenous to South Africa [29,30]. *Combretum hereroense* Schinz (russet bushwillow) is a deciduous shrub from the Combretaceae family, commonly found in Southern and Eastern Africa [25,26,31,32]. *Olea europaea* subsp. *africana* (Mill.), commonly known as the African wild olive and a member of the Oleaceae family, typically grows in diverse environments such as woodlands, riverbanks, and rocky slopes. This plant is widely distributed in Africa, China, Arabia, the Mascarene Islands, and India [33].

Previous research has shown that *B. saligna* extracts exhibit various biological activities, such as antioxidant and antiproliferative effects [34], as well as anti-inflammatory, antibiofilm, antidiabetic, and antimycobacterial properties [22,35,36]. Similarly, *C. hereroense* has been reported to possess antibacterial, antimycobacterial, and antifungal activities, primarily derived from its leaves [23]. Several bioactive compounds isolated from *C. hereroense* have shown effectiveness against mycobacteria and Gram-positive and Gram-negative bacteria [24]. Additionally, extracts from *Olea europaea* subsp. *africana* have demonstrated antibacterial, antifungal, antioxidant [37], antiviral, and antiparasitic activities [38]. Numerous compounds with broad-spectrum activity have been isolated mainly from the leaves of this plant [39].

Previous antibacterial evaluation of extracts from *B. saligna*, *C. hereroense*, and *O. europaea* subsp. *africana* have largely focused on Gram-positive and Gram-negative bacteria, with limited research into their antimycobacterial potential despite their traditional uses in treating ailments such as TB. In addition, the effect of the plant species on Mycobacteria adaptive strategies such as biofilm formation and sliding motility is underexplored. This study therefore evaluated the antimycobacterial potential of stem acetone extracts of these plants against *Mycobacterium smegmatis*, a fast-growing, non-pathogenic surrogate for *M. tuberculosis*. *M. smegmatis* is widely used in preliminary drug-screening studies due to its safety, rapid growth, cost-effectiveness, and genetic similarity to *M. tuberculosis*, eliminating the need for biosafety level 3 facilities [40,41]. Acetone was selected as the extractant for its intermediate polarity, enabling efficient recovery of diverse bioactive compounds, including polyacetylenes, terpenoids, and carboxylic acids [42,43,44]. The observed bioactivity therefore reflects the pharmacological potential of plant secondary metabolites, whose composition is influenced by genetic, environmental, and extraction-related factors [45,46,47]. The study investigated the effects of the these extracts on planktonic growth, biofilm formation, metabolic activity, and sliding motility, key adaptive mechanisms associated with mycobacterial persistence, virulence, and drug tolerance.

## 2. Materials and Methods

### 2.1. Chemicals and Reagents

The current study employed the following analytical-grade chemicals and reagents: bacteriological agar (LabM, Heywood, UK); 5 mM ammonium formate in acetonitrile (Inqaba Biotec, Tshwane, South Africa); acetone, methanol, aluminum chloride, Folin–Ciocalteu, trichloroacetic acid, sodium nitrite, sodium carbonate, sodium acetate, L-ascorbic acid, hydrogen peroxide, gallic acid, ferric chloride, 2,2-diphenyl-1-picrylhydrazyl (DPPH), quercetin, middlebrook 7H9 broth, potassium ferricyanide, and p-iodonitrotetrazolium chloride (all from Merck, Darmstadt, Germany); oleic albumin dextrose catalase (OADC) supplement and glycerol (Fluka, Buchs, Switzerland); and glucose and Bradford reagent (Sigma-Aldrich, St. Louis, MO, USA). Whatman No. 1 filter paper was purchased from Sigma-Aldrich (St. Louis, MO, USA).

### 2.2. Plant Collection and Storage

The phytotherapeutic plants used in the study, *B. saligna* (UNIN 12202662), *C. hereroense* (UNIN 1220269), and *O. europaea* subsp. *africana* (UNIN 1220261), were collected in July 2024 at the Lowveld National Botanical Garden (25°26′40″ S 30°57′58″ E) in Mpumalanga, South Africa. The selection of the plant species was based on their uses in South African traditional medicine for the treatment of tuberculosis and related symptoms. Plant material was collected under formal authorization from the South African National Biodiversity Institute (SANBI) and Turfloop Research and Ethics Committee (TREC) TREC/75/2025: PG. The plant collection strictly adhered to SANBI’s biodiversity and conservation protocols, ensuring compliance with national regulations governing the sustainable use of indigenous plant species. In accordance with the botanical garden’s established ethical norms for responsible harvesting [48], the plants were collected only within the approved regions. The collected plant specimens were submitted to the Larry Leach Herbarium at the University of Limpopo (Polokwane, South Africa) for taxonomic identification, which was conducted by Dr. Bronwyn Egan, a botanist and curator of the herbarium. The stems of these plants were separated from the leaves, cut into smaller pieces, and dried in the dark for a month. Once completely dry, the plant material was ground into fine powder using a commercial blender and stored in air-tight glass containers.

### 2.3. Extraction Procedure

A total of 1 g of finely powdered stem material was subjected to solvent extraction using 10 mL of acetone (SupraSolv^®^, Darmstadt, Germany) in a shaking incubator (New Brunswick Scientific Co., Inc., Edison, NJ, USA). An exhaustive sequential extraction was performed using acetone as the solvent. For each sample, a fixed mass of dried plant material was extracted in three successive stages using the same biomass. Initially, 10 mL of acetone was added and the mixture was agitated for 30 min, after which the extract was filtered through Whatman No. 1 filter paper into pre-weighed glass vials. The plant residue was then re-extracted with a fresh 10 mL portion of acetone for 20 min, followed by filtration. A final extraction step was conducted using another 10 mL of fresh acetone for 10 min, after which the extract was again filtered. The three filtrates were pooled and the solvent was evaporated under a stream of air at room temperature. The dried extracts were subsequently reconstituted in acetone to a final concentration of 10 mg/mL for downstream analyses.

### 2.4. Phytochemical Analysis of the Crude Extracts

Liquid chromatography-mass spectrometry (LC-MS) analysis was carried out using Waters Synapt G2 Q-TOF mass spectrometer (Waters Corporation, Milford, MA, USA) following the procedure by Matotoka et al. [49]. Before analysis, the plant extract sample were centrifuged at 12,000 rpm for 10 min to remove any fine particles. The compounds in the sample were then separated using an Acquity HSS T3 column (2.1 × 150 mm). The mobile phase consisted of two solvents: solvent A (0.1% formic acid in water) and solvent B (5 mM ammonium formate in acetonitrile) (Inqaba Biotec, Tshwane, South Africa). A 5 µL sample was injected into the system, and separation was performed at a flow rate of 0.4 mL/min. The resulting mass spectra were processed and matched against an organic compound spectral database for tentative compound identification.

### 2.5. Quantification of Polyphenolic Compounds

#### 2.5.1. Total Phenolic Content

The quantity of phenolics in the acetone extracts of the selected plants was evaluated using the Folin–Ciocalteu reagent method, previously described by Tambe and Bhambar [50]. Briefly, the concentration of the plant extracts was reduced from 10 mg/mL to 5 mg/mL with 490 µL of distilled water in different test tubes. In each test tube, 0.25 mL of Folin–Ciocalteu reagent (Sigma-Aldrich^®^, St. Louis, MO, USA) and 1.25 mL of 7% aqueous sodium carbonate (Na_2_CO_3_) (Sigma-Aldrich^®^, St. Louis, MO, USA) were added to the mixtures, followed by 30 min of incubation in the dark at room temperature. The absorbances of the mixtures were determined using an ultraviolet/visible (UV/VIS) spectrophotometer (Genesys 10S UV-VIS, Menlo Park, CA, USA) at 725 nm. A similar procedure was followed to prepare the blank and standard curve, in which the mixtures consisted of water instead of plant extracts, and in the standard curve, plant extracts were replaced by various two-fold diluted concentrations of gallic acid (1.24, 0.62, 0.31, 0.16 and 0.08 mg/mL). The experiment was carried out in triplicate and independently repeated three times. The total phenolic contents was derived from the equation of the gallic acid (Sigma-Aldrich^®^, St. Louis, MO, USA) standard curve (y = 0.6918x + 0.0039, R^2^ = 0.9976). The results were expressed as milligrams of gallic acid equivalence per gram of extract (mg GAE per gram of extract).

#### 2.5.2. Total Tannin Content

The total tannin content was determined using the Folin–Ciocalteu reagent technique, following the procedure outlined by Tambe and Bhambar [50]. A 100 µL aliquot of the plant extract (10 mg/mL) was pipetted into clean test tubes and diluted with 7.5 mL of distilled water. To this mixture, 0.5 mL of Folin–Ciocalteu reagent (Sigma-Aldrich^®^, St. Louis, MO, USA) was added, followed by vortex mixing to ensure homogeneity. Subsequently, 1 mL of 35% sodium carbonate (Na_2_CO_3_) solution (Sigma-Aldrich^®^, St. Louis, MO, USA) was introduced, and the mixture was transferred into a 10 mL volumetric flask. The flask was filled to the mark with distilled water, shaken thoroughly to mix, and incubated in the dark at room temperature for 30 min. Gallic acid (Sigma-Aldrich^®^, St. Louis, MO, USA) served as the standard control in the assay (0.0625–1 mg/mL). Absorbance measurements of the samples and the standard control were recorded against the reagent blank at 725 nm using a UV/visible spectrophotometer (Genesys 10S UV–VIS, Menlo Park, CA, USA). The total tannin content was calculated based on the gallic acid standard curve (y = 0.7918x + 0.049, R^2^ = 0.9785) and was expressed as milligrams of gallic acid equivalents per gram of extract (mg GAE/g).

#### 2.5.3. Total Flavonoid Content

The flavonoid content in the acetone extracts of selected plants was quantified using the aluminum chloride colorimetric assay, following the protocol outlined by Tambe and Bhambar [50]. A 100 µL aliquot of the plant extract (10 mg/mL) was pipetted into clean test tubes and diluted with 4.9 mL of distilled water, as well as 300 µL of 5% sodium nitrite followed by incubation for 5 min (Supelco^®^, Bellefonte, PA, USA). To this mixture, 300 µL of 10% aluminum chloride (Sigma-Aldrich^®^, St. Louis, MO, USA) was added, followed by 2 mL of 1 M sodium hydroxide (Supelco^®^, Bellefonte, PA, USA) with 5 min incubation between each additon. The final volume was adjusted to 10 mL with distilled water. Quercetin (Sigma-Aldrich^®^, St. Louis, MO, USA) served as the standard control in the assay. Absorbance measurements of both the samples and the standard control were recorded against the reagent blank at 510 nm using a UV/visible spectrophotometer (Genesys 10S UV–VIS, Menlo Park, CA, USA). The total flavonoid content was calculated from the quercetin standard curve (y = 0.1129x + 0.0043) and was expressed as milligrams of quercetin equivalents per gram of extract (mg QE/g of extract).

### 2.6. Antioxidant Activity

#### 2.6.1. Free Radical Scavenging

The free radical scavenging activity of stem acetone extracts was evaluated using the 2,2-diphenyl-1-picrylhydrazyl (DPPH) assay, following the method described by Chigayo et al. [51]. Sample concentrations, including standard control (L-ascorbic acid) (Sigma-Aldrich^®^, St. Louis, MO, USA), ranged from 15.63 to 250 µg/mL. The control solution was prepared by mixing 1 mL of distilled water with 2 mL of 0.2 mmol/L of DPPH (Sigma-Aldrich^®^, St. Louis, MO, USA). The absorbance readings for both the samples and the standard control were measured at 517 nm using a UV–visible spectrophotometer (Genesys 10S UV–VIS, Menlo Park, CA, USA), with the reagent blank consisting of 1 mL of methanol (SupraSolv^®^, Darmstadt, Germany) and 1 mL of 0.2 mmol/L DPPH. The percentage inhibition of DPPH radicals was calculated using the following formula:

$$\%\;Scavenging\;activity=\frac{Ac-As}{Ac}\times 100$$

Key: *Ac* = absorbances of the control solution, *Ac* = absorbance of the test solution. The EC_50_ was calculated using GraphPad Prism 9.

#### 2.6.2. Ferric Reducing Power

The ferric reducing power of the plant extracts was determined using a potassium ferricyanide solution, following the protocol established by Oyaizu [52] with slight modifications. L-ascorbic acid served as the standard control. Absorbance readings for both the plant extracts and the standard control were recorded at 700 nm using a UV–visible spectrophotometer (Genesys 10S UV-VIS, Menlo Park, CA, USA), with the blank reagent containing acetone in place of the plant extracts. The half-maximal effective concentration (EC_50_) values were calculated using GraphPad Prism 9.

### 2.7. Antimycobacterial Activity

#### 2.7.1. Bacterial Culture and Maintenance

The *M. smegmatis* test culture (ATCC 1441) was maintained at 4 °C on Middlebrook 7H9 agar medium supplemented with glycerol (Sigma-Aldrich^®^, St. Louis, MO, USA), Tween 80 (Sigma-Aldrich^®^, St. Louis, MO, USA) and OADC. The microbial stock culture was prepared by subculturing a single bacterial colony from a slant into 20 mL of sterile Middlebrook 7H9 broth medium supplemented with 10× diluted OADC from manufacturer formulation, 0.2% glycerol (Fluka, Buchs, Switzerland), and 0.05% Tween 80. The culture was incubated at 37 °C for 24 h with agitation at 120 rpm.

#### 2.7.2. Broth Microdilution Assay

The antimycobacterial activity of the plant extracts was assessed by determining their minimum inhibitory concentrations (MICs) against *Mycobacterium smegmatis*, following the protocol established by Eloff [18,53,54] with modifications. Rifampicin (gram/volume in 2.5% Dimethyl sulfoxide) (Sigma-Aldrich^®^, St. Louis, MO, USA) served as the standard control. Untreated bacterial culture, DMSO, acetone and wells containing only the plant extract and INT were used as negative controls. Cultures in the exponential growth phase (OD_600_ = 0.8–0.9) were used for the assay. Each plant extract (10 mg/mL) was serially diluted 2-fold in 100 µL of 7H9 Middlebrook broth in round-bottom 96-well microtiter plates. For rifampicin, 1 mg/mL was serially diluted in the same manner. An equal volume (100 µL) of *M. smegmatis* culture (at 5× 10^5^ CFU/mL) was added to each well, and plates were incubated at 37 ° C for 24 h. Growth inhibition was evaluated by adding 40 µL of the microbial growth indicator p-iodonitrophenyl tetrazolium violet (0.2 mg/mL) (Sigma-Aldrich^®^, St. Louis, MO, USA) to each well, followed by incubation at 37 °C for 30 min. The wells that remained clear indicated inhibition of bacterial growth, and the MIC was determined as the lowest concentration at which no visible growth was observed.

#### 2.7.3. Growth Curve Kinetic Assay

The impact of plant extracts on the growth kinetics of *M. smegmatis* was evaluated over a 24 h period at inhibitory (MIC) and sub-inhibitory (½ × MIC) concentrations of the plant extracts, and/using rifampicin (Sigma-Aldrich^®^, St. Louis, MO, USA) as the standard control at its MIC. The procedure followed was based on the protocol by da Silva [55] with modifications. Color controls for each extract were included to account for any interference from the natural pigmentation of plant extracts in UV–visible spectrophotometric measurements (Genesys 10S UV–VIS, Menlo Park, CA, USA). Growth was monitored at regular intervals, with samples collected every 3 h during the first 9 h and every 6 h thereafter until the 24 h mark from a culture with starting OD_600_ of 0.02 (1.11 × 10^4^ CFU/mL). This was performed for all test plant extracts, rifampicin, and color controls. The optical density at 600 nm (OD_600_) of the diluted test samples and color controls was measured. To correct color interference, the OD of the color controls was subtracted from the corresponding test samples, and the resulting values were used to construct growth kinetic curves.

### 2.8. Antibiofilm Activity Analysis (Biomass)

The impact of plant extract on biofilms formed by *M. smegmatis* was evaluated by examining its ability to prevent biofilm development, inhibit initial bacterial adhesion, and eradicate established biofilms, following the methodology described by Sandasi et al. [56,57].

#### 2.8.1. Prevention of Biofilm Formation

The inhibitory effect of plant extracts on the formation of biofilms by *M. smegmatis* was evaluated using flat bottom 96-well microtiter plates, following the protocol described by Famuyide et al. [58]. Extracts were serially diluted in equal volumes of 7H9 Middlebrook medium (Sigma-Aldrich^®^, St. Louis, MO, USA), similarly to the microbroth dilution method, but without Tween 80 in the broth. A standardized *M. smegmatis* culture (OD_600_ = 0.02) (1.11 × 10^4^ CFU/mL) was added to each well. The untreated culture was used as a control. The plates were then incubated at 37 °C without agitation for 24 h to allow biofilm development. After incubation, biofilm biomass was quantified using the crystal violet staining assay.

#### 2.8.2. Inhibition of Initial Cell Attachment

To assess the impact of plant extracts on the inhibition of initial cell attachment, biofilms were first allowed to adhere to the surfaces of 96-well microtiter plates for 4 h at 37 °C without agitation. Following this adhesion period, the wells were treated with equal volumes of plant extracts and incubated for an additional 24 h at 37 °C. The biofilm biomass was then quantified using the crystal violet staining method, as described below.

#### 2.8.3. Eradication of Mature Biofilms

The impact of plant extracts on the eradication of mature biofilms was assessed by first allowing mycobacterial biofilms to form and mature on the surfaces of 96-well microtiter plates for a 24 h incubation period at 37 °C without agitation. Subsequently, the mature biofilms were treated with equal volumes of plant extracts and incubated for an additional 24 h at 37 °C. The biofilm biomass was then quantified using the crystal violet staining method, as described below.

#### 2.8.4. Crystal Violet Staining Assay

The crystal violet staining method, originally described by Sandasi et al. [56], quantifies the biomass of mycobacterial biofilms. Microtiter plates from all biofilm developmental stages were first washed three times with sterile phosphate-buffered saline (PBS, pH 7.4) (HycloneTM, Marlborough, MA, USA) to eliminate residual planktonic cells. The plates were then air-dried and heat-fixed by incubating at 60 °C for 45 min. Subsequently, each well was stained with 0.1% (*w*/*v*) crystal violet solution (Sigma-Aldrich^®^, St. Louis, MO, USA) and incubated at room temperature for 15 min. Excess stain was removed by washing the plates three times with sterile PBS. Prior to absorbance measurement, the wells were destained using methanol to solubilize the bound crystal violet. Absorbance was recorded at 590 nm using a multiplate reader (Thermo Scientific, CAT:1530, Multiskan Sky, Singapore). The percentage of biofilm inhibition was calculated using the formula:

$$Antibiofilm\;activity=\frac{Ac-As}{Ac}\times 100$$

where *Ac* represents the absorbance of the control and *As* represents the absorbance of the sample.

#### 2.8.5. Visualization of Biofilms

To visualize mycobacterial biofilms during the prevention stage, biofilms were allowed to adhere to the surface of sterile glass cover slips in the presence of plant extracts for 24 h at 37 °C. The cover slips were gently washed three times with sterile phosphate-buffered saline (PBS) to remove non-adherent cells, then air-dried and heat-fixed by flaming. The biofilms were then stained with crystal violet for 15 min. After staining, biofilm biomass was assessed by mounting the stained cover slips on glass slides with the biofilm surface facing upward. Microscopic examination was performed using a light microscope (Labotec, Johannesburg, South Africa) at 40× magnification.

#### 2.8.6. Metabolic Activity Screening

The impact of plant extracts on the metabolic activity of mycobacterial biofilms was assessed using a modified version of the previously described antibiofilm assay. The non-adherent bacterial cells were carefully removed, and the wells were washed with sterile phosphate-buffered saline (PBS) (HycloneTM, Marlborough, MA, USA). A solution of 0.2 mg/mL iodonitrotetrazolium chloride was then added to each well to stain metabolically active (viable) cells, followed by incubation at room temperature for 15 min. The absorbance was measured at 490 nm using a multiplate reader (Thermo Scientific, CAT:1530, Multiskan Sky, Singapore). The percentage inhibition of metabolic activity was calculated using the same formula as applied in the crystal violet staining assay.

### 2.9. Anti-Sliding Motility Screening

The anti-sliding motility effects of plant extracts against *M. smegmatis* were evaluated following the protocol described by Martínez et al. [59] with slight modifications. Soft Middlebrook agar was prepared by supplementing Middlebrook medium with 0.05% (volume/volume in distilled water) Tween 80 and 0.5% bacteriological agar (LabM, Heywood, UK). The agar was infused with plant extracts at their minimum inhibitory concentration (MIC), and half MIC (½ MIC) values prior to solidification. Once solidified, the agar plates were centrally inoculated with 10 µL of an exponential phase culture (OD_600_ = 0.9) (5 × 10^5^ CFU/mL) of *M. smegmatis*, followed by incubation for 24 h at 37 °C. After incubation, the diameter of the colony spread from the point of inoculation was measured. Anti-sliding motility activity was calculated using the following formula:

$$Anti-sliding\;motility=\frac{Dcontrol-Dtreatment}{Dcontrol}\times 100$$

where *Dcontrol* is the diameter of the colony in the untreated control, and *Dtreatment* is the diameter in the presence of plant extracts.

### 2.10. Statistical Analysis

Data are presented as mean values ± standard deviation based on three independent experiments. Statistical differences between experimental groups were evaluated using two-way ANOVA followed by Dunnett’s multiple comparison test, performed on GraphPad Prism (version 9). A *p*-value less than 0.05 was considered statistically significant, while values greater than 0.05 were deemed nonsignificant.

## 3. Results

### 3.1. Phytochemical Analysis of the Crude Extracts

**Figure 1:**
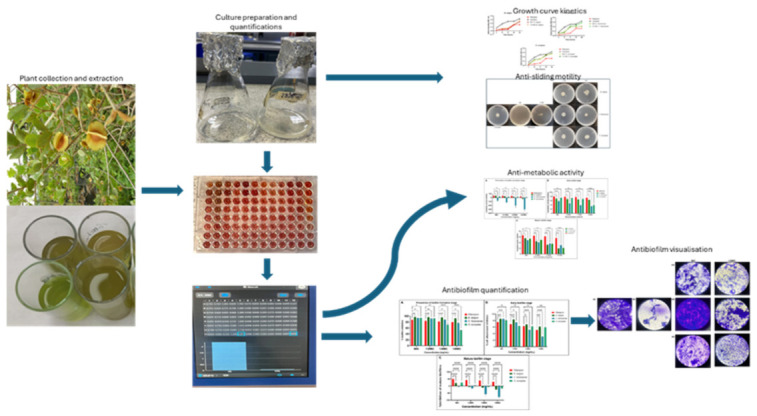
A schematic figure of the overall study workflow followed to achieve the aim of the study.

**Figure 2:**
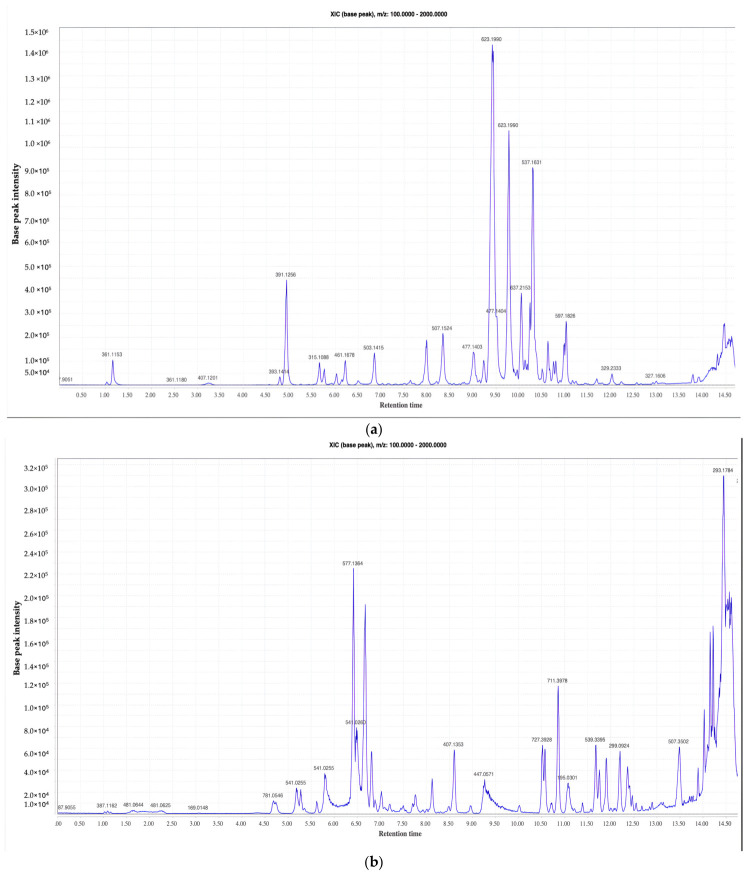

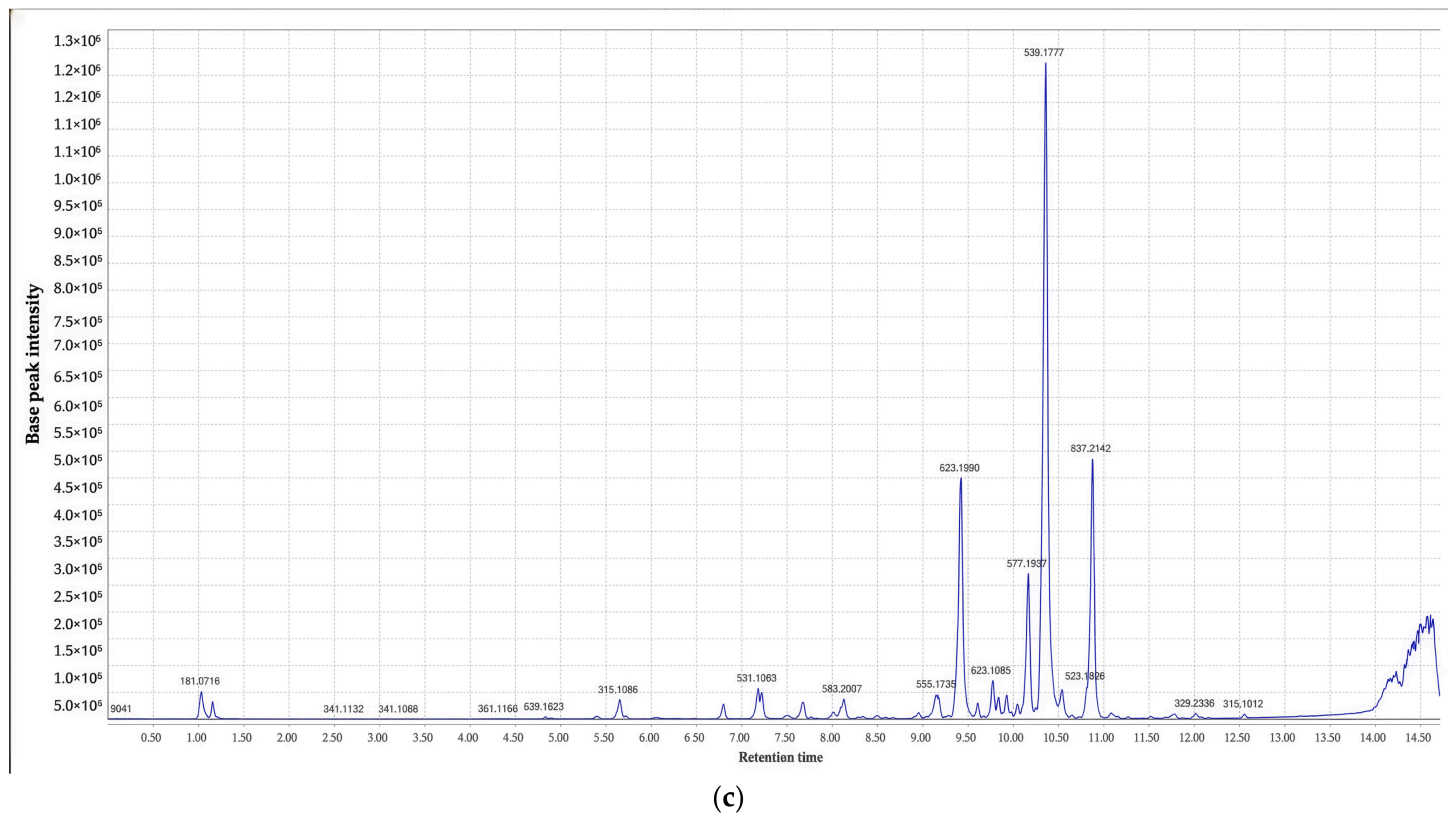
Liquid chromatography–mass spectroscopy chromatogram spectra of stem acetone extracts of *B. saligna* (**a**), *C. hereroense* (**b**), and *O. europaea* subsp. *africana* (**c**).

The phytochemicals from the selected acetone extracts were identified using LCMS and their biological activities were investigated by evaluating antimycobacterial, antibiofilm, and anti-motility (Figure 1). The chromatogram of *B. saligna* showed a moderately complex profile, characterized by a cluster of prominent peaks between 9.0 and 11.0 min (Figure 2a). In contrast, *C. hereroense* exhibited the highest chemical complexity, with a dense cluster of peaks between 5.5 and 7.0 min and a broad distribution of signals across the chromatographic range (5–13 min) (Figure 2b). The LC–MS profile of *O. europaea* was comparatively less complex but dominated by a major peak at approximately 10.7 min, accompanied by several secondary peaks within the 9.0–11.5 min range (Figure 2c). While all three extracts exhibited major metabolite elution within the 9–11 min window, *C. hereroense* demonstrated the greatest chemical diversity, *B. saligna* showed an intermediate profile, and *O. europaea* was characterized by fewer but relatively high-abundance constituents.

LC–MS-based tentative identification showed that all three plant extracts are rich in polyphenolic compounds, mainly phenolic acids, flavonoids, and their glycosides. B. saligna was characterized by the presence of phenolic acids such as gentisic acid 5-O-β-glucoside, glucosyringic acid, and chlorogenic acid, together with procyanidin dimers (B1–B3) and flavonoids including orientin, acteoside, rosmarinic acid, and quercetin (Table 1). C. hereroense exhibited the highest chemical diversity, containing hydrolysable tannins (punicalin and corilagin), procyanidin oligomers, and a wide range of flavonoid glycosides such as kaempferol-3-O-glucoside, nicotiflorin, diosmin, and hesperidin, as well as later-eluting flavonoid aglycones including isokaempferide and eupatilin. O. europaea subsp. africana was characterized by phenolic acids (neochlorogenic and chlorogenic acids), the secoiridoid oleuropein, and multiple flavonoid glycosides such as apigenin-7-O-glucoside, naringin, hesperidin, and diosmin, along with flavonoid aglycones at higher retention times.

### 3.2. Quantitative Phytochemical Analysis and Antioxidant Activity

The highest total phenolic content was from *O. europaea* subsp. *africana* (27.18 ± 0.77 mgGAE/g extract) extracts, while *B. saligna* had the highest total tannin (287.18 ± 0.19 mgGAE/g extract) and flavonoid (16.48 ± 0.05 mgQE/g extract) contents (Table 2). The antioxidant activities were expressed as EC_50_ values. These values indicate the concentration of plant extract that is required to reduce 50% of free radicals in a solution. Therefore, lower EC_50_ values reflect stronger antioxidant potential. Ascorbic acid served as the reference standard in both assays. Among the tested extracts, *B. saligna* (17.66 ± 5.396 and 399.1 ± 3.717 µg/mL) exhibited the highest antioxidant activity in both tests, while *C. hereroense* (32.16 ± 6.495 and 2238 ± 0.9706 µg/mL) and *O. europaea* subsp. *africana* (57.84 ± 4.888 and 13.934 ± 1.439 µg/mL) displayed the weakest effects in both assays when compared to that of *B. saligna*.

### 3.3. Antimycobacterial Activity

#### 3.3.1. Broth Microdilution Assay

The antimycobacterial activity of stem acetone extracts was expressed as minimum inhibitory concentration (MIC) (Table 3). MIC is the lowest concentration of the extracts that can inhibit the growth of *M. smegmatis*, with values below 2.5 mg/mL considered potent. All plant extracts demonstrated strong activity, showing MICs of 0.31 mg/mL (*B. saligna*), 0.16 mg/mL (*C. hereroense*), 0.63 mg/mL (*O. europaea* subsp. *africana*), and 0.08 mg/mL for rifampicin.

#### 3.3.2. Growth Curve Kinetics

**Figure 3:**
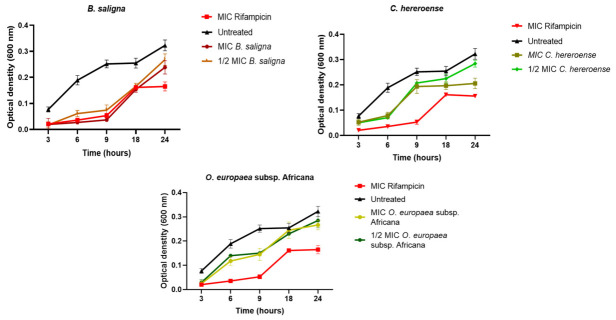
Effect of the stem acetone plant extracts from *B. saligna*, *C. hereroense* and *O. europaea* subsp. *africana* (MIC, ½ × MIC) and rifampicin (MIC) on the growth kinetics of *M. smegmatis* monitored at different time points at 600 nm. MIC: Minimum inhibitory concentration. All values are presented as means of triplicate ± standard deviation (SD).

The plant extracts exhibited bacteriostatic effect on *M. smegmatis* with significant activity from the *B. saligna* and *C. hereroense* extracts (Figure 3). *B. saligna* extract produced the prolonged lag phase, which lasted for approximately 9 h, followed by *C. hereroense* extract, which prolonged the lag phase about 6 h. The growth curve of *B. saligna* was most comparable to that of the standard control (rifampicin). The bacteriostatic activity was indicated by a pronounced extension of the lag phase, suggestive of an inhibition of key metabolic processes required for cell cycle progression. However, with further incubation, the bacterial cells resumed rapid division and transitioned promptly into the exponential growth phase, similar to the untreated culture. This showed that the initial growth inhibition was bacteriostatic. Conversely, exposure to *O. europaea* subsp. *africana* did not induce a measurable prolongation of the lag phase, implying minimal interference with early adaptive or preparatory growth mechanisms.

### 3.4. Evaluation of Antibiofilm Activity

#### 3.4.1. Antibiofilm Biomass Activity

**Figure 4:**
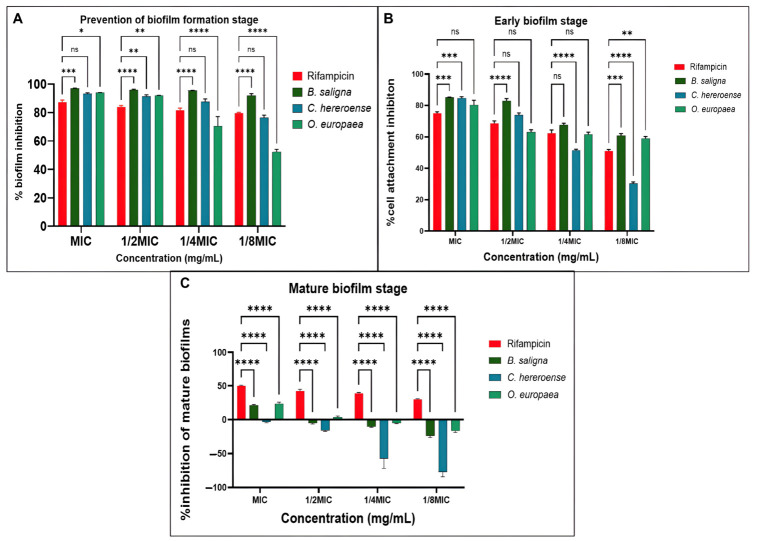
Biofilm inhibition activity of the plant extracts from *B. saligna*, *C. hereroense* and *O. europaea* subsp. *africana* against *M. smegmatis* targeted at the biofilm formation stage (**A**), early biofilm stage (**B**), and mature biofilm stage (**C**). Rifampicin served as the positive control. MIC: Minimum inhibitory concentration. All values are presented as mean ± standard deviation (SD) of duplicates from two separate tests. Two-way analysis of variance (ANOVA) coupled with Dunnett’s multiple comparison test was used to assess the statistical significance of the differences. (ns): not significant; (*): *p* < 0.05; (**): *p* < 0.01; (***): *p* < 0.001; (****): *p* < 0.0001.

Inhibition rates above 50% in the antibioflm biomass activity assay were considered significant and values below 0% indicated biofilm enhancement (Figure 4). In the prevention of biofilm formation stage (Figure 4A), all the plant extracts together with rifampicin demonstrated more than 50% inhibition across all tested concentrations. Similarly, the plant extracts inhibited more than 50% of the preformed biofilms across all concentrations except for *C. hereroense* at $\frac{1}{8}$ mg/mL (Figure 4B). The eradication of the mature biofilm stage (Figure 4C) was the challenging phase for all the plant extracts, while rifampicin obtained more than 50% inhibition of the mature biofilm only at its MIC. The antibiofilm activity of all the plant extracts was less than 50%, with enhancement of biofilm formation as the concentration of the extracts reduced.

#### 3.4.2. Visualization of Biofilm Initial Attachment Prevention

**Figure 5:**
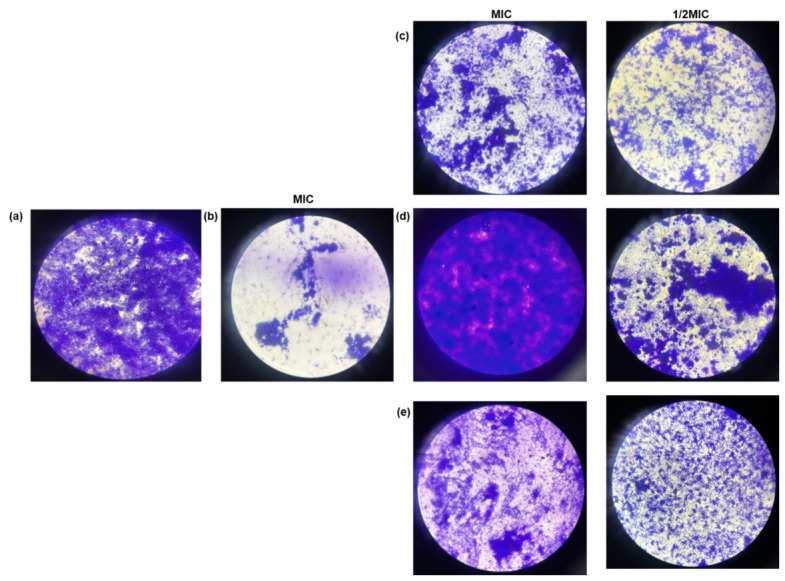
Visualization of the prevention of biofilm initial cell attachment by acetone plant extracts from *B. saligna* (**c**), *C. hereroense* (**d**), and *O. europaea* subsp. *africana* (**e**). The untreated control (**a**) represents biofilm cells with no therapeutic treatment. Rifampicin (**b**) served as the positive control at its MIC. MIC: Minimum inhibitory concentration.

Rifampicin at its MIC served as the positive control, while untreated *M. smegmatis* cells represented the baseline biofilm formation (Figure 5). The untreated cells (a) exhibited extensive surface attachment, forming a dense biofilm network on the cover slips. The treatment with rifampicin (b), *B. saligna* (c), *C. hereroense* (d), and *O. europaea* subsp. *africana* (e) resulted in visibly reduced microbial colonization, indicating varying degrees of inhibition of initial surface attachment.

#### 3.4.3. Metabolic Activity

**Figure 6:**
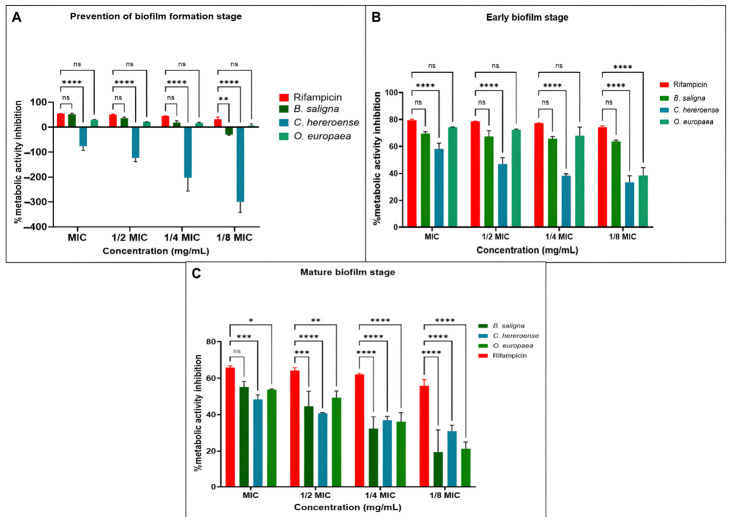
Effects of stem acetone extracts from *B. saligna*, *C. hereroense* and *O. europaea* subsp. *africana* on metabolism within *M. smegmatis* biofilms targeted at the biofilm formation stage (**A**), early biofilm stage (**B**), and mature biofilm stage (**C**). Rifampicin served as the positive control. MIC: Minimum inhibitory concentration. All values are presented as mean ± standard deviation (SD). Two-way analysis of variance (ANOVA) coupled with Dunnett’s multiple comparison test was used to assess the statistical significance of the differences. (ns): not significant; (*): *p* < 0.05; (**): *p* < 0.01; (***): *p* < 0.001; (****): *p* < 0.0001.

Inhibition rates above 50% in the metabolic activity assay was considered significant and values below 0% indicated enhancement of metabolic activity (Figure 6). The extracts showed less inhibition and enhancement of the metabolic activity at the prevention of the biofilm formation stage (Figure 6A). Significant inhibition of the metabolic activity was observed at the early biofilm and mature biofilm stages from all extracts (Figure 6B,C). Rifampicin maintained a significant inhibition of the metabolic activities across all stages of biofilm formation.

### 3.5. Anti-Sliding Motility

**Figure 7:**
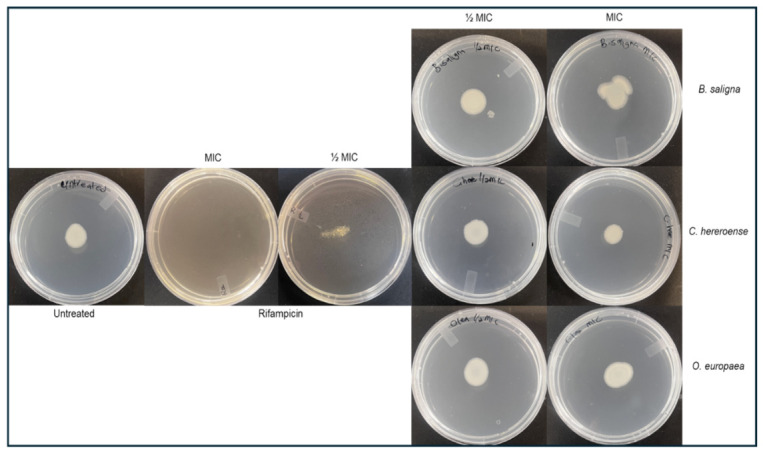
Sliding motility of *M. smegmatis* after treatment with stem acetone plant extracts.

The sliding motility was indicated by the increase in layers around the point of inoculation after the 24 h period (Table 3 and Figure 7). Only rifampicin showed significant anti-sliding motility at both its MIC (100%) and ½ × MIC (75%). The anti-sliding motility of all the extracts was nonsignificant (less than 50%), with *B. saligna* and *O. europaea* subsp. African extracts influenced the enhancement of sliding motility.

## 4. Discussion

The increasing resistance of Mycobacteria to conventional antibiotics necessitates alternative therapeutics. In this study, plant extracts demonstrated preliminary antimycobacterial activity against Mycobacterium smegmatis (MICs), which may be attributed to the combined action of multiple polphenolic compounds through additive and/or synergistic interactions. Consequently, it would be challenging to attribute the observed biological activity solely to a single compound within the crude extracts [60,61,62].

LC-MS analysis of the plant extracts revealed that a total of 28 phytochemical compounds were identified in the *B. saligna* extract, which was predominantly enriched in flavonoids. In comparison, 48 compounds were detected in both *C. hereroense* and *O. europaea* subsp. *africana*, respectively, with these extracts showing a predominance of proanthocyanidins and flavonoid glycosides (Table 1). The observed dominance of specific phytochemical classes among the different plant species suggested potential variation in their therapeutic properties. Flavonoids and flavonoid glycosides have been widely reported to exhibit antioxidant, anti-inflammatory, and antimicrobial activities [63,64], while proanthocyanidins are similarly associated with strong antioxidant, anti-inflammatory, and antibacterial effects [65,66]. Among the three species, *B. saligna* showed the highest concentrations of total tannins (287.18 ± 0.19 mg GAE/g extract) and flavonoids (16.48 ± 0.05 mg QE/g extract). In contrast, *O. europaea* subsp. *africana* extracts had the highest total phenolic content (27.18 ± 0.77 mg GAE/g of extract). Although *C. hereroense* exhibited the lowest total flavonoid concentration (1.79 ± 0.13 mgQE/g extract), its tannin levels were closely comparable to those of *B. saligna* (245.45 ± 0.36). Flavonoids in *C. hereroense* are known to form condensed tannins, including gallotannins and ellagitannins, which occur abundantly in traditional medicinal preparations derived from *Combretum* species [67,68]. The presence of polyphenols and flavonoids in *B*. *saligna* has also been reported in previous studies by Adedapo et al. [36] and Nina et al. [22]; however, differences in the extraction solvents used may have contributed to the variation in the phytochemical profiles and results obtained in the present study. *O. europaea* subsp. *africana* has previously been reported to contain tannins, terpenoids, flavones, flavonoids, alkaloids, and steroids in leaf extracts obtained using solvents of different polarity by Adem et al. [37]. Similarly, Mukesi et al. [69] identified biophenols, benzoic acid derivatives, secoiridoids, and sterols through GC-MS analysis.

Mycobacterial infections are associated with the depletion of antioxidant defenses due to excessive reactive oxygen species (ROS), which leads to heightened inflammation and immune dysfunction. Agents with antioxidant activity may provide adjunct therapeutic benefits by limiting oxidative stress-induced conditions that favor mycobacterial persistence and reducing bacterial survival [15,70,71]. B. saligna exhibited the strongest antioxidant activity (17.66 ± 5.396 and 399.1 ± 3.717 µg/mL), whereas C. hereroense (32.16 ± 6.495 and 2238 ± 0.9706 µg/mL) and O. europaea subsp. africana (57.84 ± 4.888 and 13.934 ± 1.439 µg/mL) showed comparatively weaker effects, particularly in the DPPH assay (Table 2). A previous study by Twilley et al. [72] demonstrated highest antioxidant activity from the stem ethanol extracts of *B*. *saligna* as compared to the leaf extarcts. The antioxidant activity of the leaf and stem methanolic extracts of *B. saligna* had been previously demonstrated [36]. The presence of flavonoids and coumarins in medicinal plant extracts has been reported to influence their antioxidant capacity due to their ability to scavenge free radicals and chelate metal ions [73]. *O. europaea* subsp. *africana* is commonly known for its antioxidant and anticancer potential [74]. Leaf extracts of *O. europaea* subsp. *africana* have been shown to exhibit antioxidant activity through ferric reducing power capabilities primarily due to camphor and oleuropein, which have been reported to have antioxidant and antimicrobial capabilities [37]. In the present study, phytochemical analysis further revealed the presence of compounds such as rosmarinic acid, naringin, quercetin-3-O-rhamnoside, and kaempferol 3-neohesperidoside, which may significantly contribute to the observed antioxidant activity.

*C. hereroense* exhibited the highest antimycobacterial potency against *M. smegmatis*, with a MIC value of 0.16 mg/mL, followed by *B. saligna* at 0.31 mg/mL and *O. europaea* subsp. *africana* at 0.63 mg/mL. Previous studies have reported MIC values of 0.47 mg/mL [75] and 0.63 mg/mL [76] for acetone leaf extracts of *C. hereroense* against *M. smegmatis*, which are comparable to the activity observed in the present study. Notably, the MIC values obtained from stem extracts in this study underscore the therapeutic relevance of stems, which are often underexplored in both traditional applications and scientific investigations compared to leaves. Furthermore, *C. hereroense* has been widely reported to possess broad-spectrum antimicrobial activity across different plant parts. For example, aqueous root extracts have demonstrated activity against *Neisseria gonorrhoeae* [77], while leaf extracts have shown antibacterial effects against *M. smegmatis* [75], *Pseudomonas aeruginosa*, *Staphylococcus aureus*, *Escherichia coli*, and *Enterococcus faecalis* [53]. Stem extracts have also been reported to inhibit *Staphylococcus epidermidis* and *Sarcina* species [78]. In addition, Katerere et al. [24] isolated three phenolic compounds from the fruit of *C. hereroense*; 5,7-dimethoxy-1,2,3-phenanthrenetriol, 5,7-dimethoxy-2,3-phenanthrenediol, and 9,10-dihydro-3,5-dimethoxy-2,7-phenanthrene, which exhibited antimicrobial activity against *Staphylococcus aureus* and *Mycobacterium fortuitum*. Collectively, these findings, together with the results of the present study, highlight the considerable therapeutic potential of different plant parts of *C. hereroense* against a wide range of pathogenic microorganisms.

Bamuamba et al. [35] previously reported antimycobacterial activity in *B. saligna* hexane extracts, which was attributed to the presence of the triterpenoid oleanolic acid, a compound also noted for its non-cytotoxic nature.

The antibacterial properties of *O. europaea* subsp. *africana* have been predominantly investigated using leaf extracts, which have demonstrated broad-spectrum activity against several pathogens, including *Bacillus cereus*, *Enterococcus faecalis*, *Moraxella catarrhalis*, *Pseudomonas aeruginosa*, *Salmonella typhi*, and *Staphylococcus aureus* [37], as well as *Escherichia coli* [38], *Proteus vulgaris*, and *Klebsiella pneumoniae* [79]. Oleuropein, a major phenolic compound identified in these leaf extracts, has been widely recognized for its antimicrobial activity against a broad range of microorganisms, including bacteria, fungi, parasites, and viruses [33,37].

In the present study, growth curve kinetics demonstrated that at both MIC and ½ × MICs of the stem acetone extracts, as well as at rifampicin MIC, *M. smegmatis* growth was significantly inhibited for the first 9 h of incubation (Figure 3). This growth pattern suggests an extension of the lag phase, indicating that the microorganism was initially adapting to the stress imposed by the extracts, consistent with previous reports describing prolonged lag phases following exposure to antimicrobial agents [18,80,81]. Notably, the inhibition of *B. saligna* growth did not differ significantly from that of the standard control, rifampicin, highlighting the strong activity of the stem acetone extract in suppressing planktonic *M. smegmatis* growth.

In addition, *C. hereroense* elongated the lag phase by 6 h. *O. europaea* subsp. *africana* had a negligible effect on the lag phase but significantly reduced the growth rate of the culture for 9 h. The consistent reduction in microbial populations following treatment with the plant extracts highlights their potential as natural antimicrobial agents, which is likely attributable to the poplyphenolic compounds present in the stem extracts. The noticeable delay in microbial growth, reflected by an extended lag phase and slower proliferation, suggested that these polyphenolics may interfere with key physiological processes required for bacterial adaptation and replication.

Mycobacteria are responsible for over 80% of resistant and persistent infections, largely due to biofilm formation in the lungs [8,82]. These biofilms are embedded in an extracellular polymeric substance (EPS) matrix, mainly composed of exopolysaccharides that mediate cell adhesion, structural stability, and surface attachment [83]. EPS further limits antibiotic penetration through physicochemical interactions, contributing to drug tolerance [84,85], while its overproduction enhances biofilm adaptability and development [86,87]. The initial attachment of the bacterium to a surface is a reversible and loose interaction [88]. As such, the plant extracts used in the study successfully interfered with this type of interaction with notable activities (more than 50% inhibition) at all concentrations to prevent initial cell attachment (Figure 4A) and inhibit the stages of the preformed biofilms (Figure 4B).

Mature biofilms are characterized by irreversible surface attachment and extensive production of extracellular polymeric substances (EPS), particularly exopolysaccharides, which confer structural integrity and reduced permeability to antimicrobial agents [86,89]. In this study, all extracts showed limited efficacy in disrupting mature biofilms (Figure 4C). At lower concentrations, extracts even enhanced biofilm biomass, while rifampicin maintained >50% inhibition across all stages. This reduced efficacy of the plant extracts, despite their polyphenolic content, may be explained by the limited penetration of polyphenolics through the dense EPS matrix, as well as their reduced availability at the biofilm core, where bacteria are more metabolically protected and less susceptible to bacteriostatic effects. In addition, sub-inhibitory concentrations of polyphenolics may contribute to stress responses that promote EPS production and biofilm strengthening rather than disruption [90,91]. These findings highlight the importance of distinguishing between bacteriostatic and antibiofilm effects in relation to phytochemical composition. While polyphenols are known to interfere with microbial metabolism and adhesion, their activity may be significantly reduced in mature biofilms due to diffusion barriers and adaptive bacterial responses within the biofilm environment [89]. Targeting EPS synthesis and biofilm architecture is therefore critical for effective eradication rather than surface-level biomass reduction alone.

Rifampicin served as a positive control, while untreated *M. smegmatis* cells represented the formation of the baseline biofilm (Figure 5). The untreated cells exhibited extensive surface attachment, forming a dense biofilm network on the cover slips. Treatment with rifampicin (b), *B. saligna* (c), *C. hereroense* (d), and *O. europaea* subsp. *africana* (e) resulted in visibly reduced microbial colonization, indicating varying degrees of inhibition of initial surface attachment. By restricting the analysis to the early stage of biofilm formation, the study enabled a more reliable and interpretable assessment of the antibiofilm activity of the plant extracts, as this phase is more susceptible to disruption prior to the establishment of a mature, EPS-rich biofilm structure [92,93].

Assessing biofilm metabolic activity in addition to biomass is essential because biomass quantification alone does not differentiate between viable, metabolically active cells and inactive or dead cells embedded within the biofilm matrix. Metabolic activity assays therefore provide complementary insight into the physiological status and viability of biofilm-associated bacteria. During the biofilm formation stage (Figure 6A), all plant extracts demonstrated minimal inhibition of metabolic activity across all tested concentrations. Notably, *C. hereroense* appeared to enhance metabolic activity, with the degree of enhancement increasing at lower extract concentrations. At this early phase, bacterial cells are more sensitive to therapeutic treatment than their planktonic counterparts. This sensitivity could be attributed to the absence of biofilm matrix, which can assist in retaining the extracts within the biofilm for them to exert their antimicrobial activity [94]. At both the early and mature stages of biofilm development (Figure 6B,C), the plant extracts demonstrated the highest inhibition of metabolic activity. Notably, the activity of *B. saligna* showed no statistically significant difference compared to the standard control, rifampicin. This enhanced activity is likely attributed to the development of the EPS layer, which can retain bioactive compounds from the plant extracts, thereby prolonging exposure and allowing the extracts to exert antimicrobial effects on metabolically active bacterial cells. Furthermore, mature biofilms often harbor cells under oxidative stress, which compromises the integrity of microbial metabolisms. Consequently, plant extracts with antioxidant properties can significantly disrupt metabolic processes in established biofilms [94,95]. Accordingly, the pronounced inhibition of metabolic activity in *M. smegmatis* observed upon treatment with *B. saligna* may be attributed to combinational effects of phytochemicals in *B. saligna* that also exhibit antioxidant activity. However, in humans, the strong anti-metabolic activity of plant extracts may inadvertently disrupt the metabolic functions of beneficial gut microorganisms, many of which exist in biofilm communities. This potential adverse effect can be mitigated by co-administering probiotics alongside the plant extracts. Probiotics support the repopulation of the gut microbiota, promote biofilm formation, and enhance microbial diversity and metabolic resilience. When combined, plant extracts and probiotics may exhibit a synergistic effect that helps maintain a balanced and healthy gut ecosystem [96,97].

Sliding motility is a passive form of surface translocation in bacteria such as *M. smegmatis*, driven by reduced friction from self-produced surfactants, enabling colony expansion across semisolid media [10,98]. This behavior contributes to biofilm formation, persistence, and infection. On agar, sliding is observed as concentric layers radiating from the inoculation point, progressing from a monolayer to multilayered growth [12]. Inhibition >50% is considered significant, whereas negative values indicate enhanced motility. In this study, the plant extracts showed nonsignificant effects on the sliding motility of *M. smegmatis* at their MIC and ½ × MICs. At MIC concentrations, *B. saligna* and *C. hereroense* did not significantly inhibit sliding motility by 10 and 30% while *O. europaea* subsp. *africana* had no effect on sliding motility. *C. hereroense* had anti-sliding effects at ½ × MIC. At ½ × MIC concentrations, *B. saligna* and *O. europaea* subsp. *africana* enhanced sliding motility. Rifampicin showed significant inhibition of sliding motility at its ½ MIC concentration (75%) and MIC concentration (100%). A study by Tsygano and Tkachenko [99] showed the effective role of rifampicin against actively dividing cells in the sliding colony. The same author suggested that the contributing factor to the observed activities of extracts against sliding motility may be linked to the resistant characteristics of surfactants in mycobacterial cultures, such as GPL, extracellular signaling molecules. While the plant extracts showed notable antibiofilm activity during early biofilm development, the limited effect on sliding motility may reflect fundamental mechanistic differences, as sliding motility is driven by glycopeptidolipids rather than cell adhesion and extracellular matrix interactions [12,100].

## 5. Conclusions

This study demonstrated the antioxidant and antimycobacterial properties of stem acetone extracts of *B. saligna*, *C. hereroense*, and *O. europaea* subsp. *africana* against *Mycobacterium smegmatis*. The findings suggested that these extracts may be effective against planktonic cells and early-stage biofilms of *M. smegmatis*. The observed bioactivities provide preliminary screening evidence of antimycobacterial potential of the plant extracts, which may be attributed to the polyphenolic constituents of the stem parts, likely acting through additive and/or synergistic interactions.

Future research could explore additional critical mechanisms involved in biofilm formation and persistence, such as the roles of exopolysaccharides and quorum sensing. A key limitation of the present study is the absence of cytotoxicity assessments for the plant extracts, which is necessary to determine their safety profile within the antitubercular drug discovery pipeline. Moreover, bioassay-guided fractionation and optimization of the crude extracts may be required to enhance activity and isolate key bioactive compounds responsible for the observed effects.

## Figures and Tables

**Table 1:** LC-MS analysis of stem acetone extracts of *B. saligna*, *C. hereroense* and *O. europaea* subsp. *africana*.

RT (min)	IUPAC Name (Common Name)	Molecular Formula	Monoisotopic Mass	Mass Error (ppm)
	*B. saligna*			
5.26	2-hydroxy-5-[(2S,3R,4S,5S,6R)-3,4,5-trihydroxy-6-(hydroxymethyl)oxan-2-yl]oxybenzoic acid (**Gentisic acid 5-O-β-glucoside**)	C_13_H_16_O_9_	315.07	−0.50
6.14	3,5-dimethoxy-4-[(2S,3R,4S,5S,6R)-3,4,5-trihydroxy-6-(hydroxymethyl)oxan-2-yl]oxybenzoic acid (**Glucosyringic acid**)	C_15_H_20_O_10_	359.10	3.43
6.42	(2R,3S)-2-(3,4-dihydroxyphenyl)-8-[(2R,3R,4R)-2-(3,4-dihydroxyphenyl)-3,5,7-trihydroxy-3,4-dihydro-2H-chromen-4-yl]-3,4-dihydro-2H-chromene-3,5,7-triol (**Procyanidin B1**)	C_30_H_26_O_12_	577.14	−4.74
6.46	6,7-Dihydroxycoumarin-6-glucoside (**Esculin**)	C_15_H_16_O_9_	339.07	1.73
6.52	(2R,3R)-2-(3,4-dihydroxyphenyl)-8-[(2R,3R,4R)-2-(3,4-dihydroxyphenyl)-3,5,7-trihydroxy-3,4-dihydro-2H-chromen-4-yl]-3,4-dihydro-2H-chromene-3,5,7-triol (**Procyanidin B2**)	C_30_H_26_O_12_	577.14	3.45
6.56	(2R,3S,4S,4aR,10bS)-3,4,8,10-tetrahydroxy-2-(hydroxymethyl)-9-methoxy-3,4,4a,10b-tetrahydro-2H-pyrano[3,2-c]isochromen-6-one (**Bergenin**)	C_14_H_16_O_9_	327.07	3.80
6.64	(1R,3R,4S,5R)-1,3,4-trihydroxy-5-[(E)-3-(4-hydroxyphenyl)prop-2-enoyl]oxycyclohexane-1-carboxylic acid (**3-p-Coumaroylquinic acid**)	C_16_H_18_O_8_	337.09	2.25
7.02	(1S,3R,4R,5R)-3-[(E)-3-(3,4-dihydroxyphenyl)prop-2-enoyl]oxy-1,4,5-trihydroxycyclohexane-1-carboxylic acid (**Chlorogenic acid**)	C_16_H_18_O_9_	359.09	2.37
7.17	(2S,3R,4S,5S,6R)-2-[4-[4-[hydroxy-(4-hydroxy-3-methoxyphenyl)methyl]-3-(hydroxymethyl)oxolan-2-yl]-2-methoxyphenoxy]-6-(hydroxymethyl)oxane-3,4,5-triol (**cycloolivil 6-O-beta-D-glucopyranoside**)	C_26_H_34_O_12_	537.20	−2.39
7.35	(2S,3S,4R,5R)-4-hydroxy-2,5-bis(hydroxymethyl)-2-[(2R,3R,4S,5S,6R)-3,4,5-trihydroxy-6-(hydroxymethyl)oxan-2-yl]oxyoxolan-3-yl] (E)-3-(4-hydroxy-3,5-dimethoxyphenyl)prop-2-enoate (**Sibiricose A6**)	C_23_H_32_O_15_	547.17	0.80
7.57	(2R,3R)-2-(3,4-dihydroxyphenyl)-3,4-dihydro-2H-chromene-3,5,7-triol (**Epicatechin**)	C_15_H_14_O_6_	290.27	−1.33
7.84	1,3,6,7-tetrahydroxy-2-[(2S,3R,4R,5S,6R)-3,4,5-trihydroxy-6-(hydroxymethyl)oxan-2-yl]xanthen-9-one (**Mangiferin**)	C_19_H_18_O_11_	421.08	3.40
7.99	[(2R,3S,4S,5R,6R)-6-[(2S,3S,4S,5R)-3,4-dihydroxy-2,5-bis(hydroxymethyl)oxolan-2-yl]oxy-3,4,5-trihydroxyoxan-2-yl]methyl (E)-3-(4-hydroxy-3,5-dimethoxyphenyl)prop-2-enoate (**Arillatose B**)	C_22_H_30_O_14_	517.16	4.28
8.09	(2R,3S)-2-(3,4-dihydroxyphenyl)-8-[(2R,3S,4S)-2-(3,4-dihydroxyphenyl)-3,5,7-trihydroxy-3,4-dihydro-2H-chromen-4-yl]-3,4-dihydro-2H-chromene-3,5,7-triol (**Procyanidin B3**)	C_30_H_26_O_12_	577.13	−1.03
8.33	(2S,3R,4S)-2-[(2S,3R,4S,5S,6R)-3-[(E)-3-(3,4-dihydroxyphenyl)prop-2-enoyl]oxy-4,5-dihydroxy-6-(hydroxymethyl)oxan-2-yl]oxy-3-ethenyl-4-(2-hydroxyethyl)-3,4-dihydro-2H-pyran-5-carboxylic acid	C_25_H_30_O_13_	537.16	4.61
8.67	2-(3,4-dihydroxyphenyl)-5,7-dihydroxy-8-[(2S,3R,4R,5S,6R)-3,4,5-trihydroxy-6-(hydroxymethyl)oxan-2-yl]chromen-4-one **(Orientin)**	C_21_H_20_O_11_	447.09	2.73
9.01	[(2R,3S,4R,5R,6R)-6-[2-(3,4-dihydroxyphenyl)ethoxy]-4,5-dihydroxy-2-(hydroxymethyl)oxan-3-yl] (E)-3-(3,4-dihydroxyphenyl)prop-2-enoate **(Calceolarioside A)**	C_23_H_26_O_11_	477.14	0.46
9.01	(2S,3R,4S,5S,6R)-2-[4-[(3S,3aR,6R,6aS)-6-[3,5-dimethoxy-4-[(2S,3R,4S,5S,6R)-3,4,5-trihydroxy-6-(hydroxymethyl)oxan-2-yl]oxyphenyl]-1,3,3a,4,6,6a-hexahydrofuro[3,4-c]furan-3-yl]-2,6-dimethoxyphenoxy]-6-(hydroxymethyl)oxane-3,4,5-triol (**Eleutheroside E**)	C_34_H_46_O_18_	741.26	4.60
9.37	2-(3,4-dihydroxyphenyl)-5,7-dihydroxy-3-[(2S,3R,4S,5R)-3,4,5-trihydroxyoxan-2-yl]oxychromen-4-one (**Reynoutrin**)	C_20_H_18_O_11_	434.3	−7.61
9.77	[(2R,3R,4R,5R,6R)-6-[2-(3,4-dihydroxyphenyl)ethoxy]-5-hydroxy-2-(hydroxymethyl)-4-[(2S,3R,4R,5R,6S)-3,4,5-trihydroxy-6-methyloxan-2-yl]oxyoxan-3-yl] (E)-3-(3,4-dihydroxyphenyl)prop-2-enoate (**Acteoside**)	C_29_H_36_O_15_	623.20	3.45
9.99	(2R)-3-(3,4-dihydroxyphenyl)-2-[(E)-3-(3,4-dihydroxyphenyl)prop-2-enoyl]oxypropanoic acid (**Rosmarinic acid**)	C_18_H_16_O_8_	359.08	−1.00
10.23	3,5,7-trihydroxy-2-[3-hydroxy-4-[(2S,3R,4S,5S,6R)-3,4,5-trihydroxy-6-(hydroxymethyl)oxan-2-yl]oxyphenyl]chromen-4-one (**Spiraeoside**)	C_21_H_20_O_12_	463.09	−0.23
10.58	3-[(2S,3R,4S,5S,6R)-4,5-dihydroxy-6-(hydroxymethyl)-3-[(2S,3R,4R,5R,6S)-3,4,5-trihydroxy-6-methyloxan-2-yl]oxyoxan-2-yl]oxy-2-(3,4-dihydroxyphenyl)-5,7-dihydroxychromen-4-one (**Quercetin 3-neohesperidoside**)	C_27_H_30_O_16_	609.14	−2.46
10.78	7-hydroxy-2-(4-hydroxy-3,5-dimethoxyphenyl)-5-[(2S,3R,4S,5S,6R)-3,4,5-trihydroxy-6-(hydroxymethyl)oxan-2-yl]oxychromen-4-one (**Tricin 5-glucoside**)	C_23_H_24_O_12_	491.12	−1.42
10.80	(3S,7R)-11-methoxy-6,8,16,20-tetraoxapentacyclo[10.8.0.02,9.03,7.013,18]icosa-1,9,11,13(18)-tetraene-17,19-dione (**Aflatoxin G2**)	C_17_H_14_O_7_	329.07	0.85
10.86	[(2S,3R,4S,5S,6R)-3,4,5-trihydroxy-6-(hydroxymethyl)oxan-2-yl] (1R,2R,4aS,6aR,6aS,6bR,8aR,9R,10R,11R,12aR,14bS)-1,10,11-trihydroxy-9-(hydroxymethyl)-1,2,6a,6b,9,12a-hexamethyl-2,3,4,5,6,6a,7,8,8a,10,11,12,13,14b-tetradecahydropicene-4a-carboxylate (**Dotorioside II**)	C_36_H_58_O_11_	666.80	1.99
10.87	2-(3,4-dihydroxyphenyl)-3,5,7-trihydroxychromen-4-one (**Quercetin**)	C_15_H_10_O_7_	301.03	−0.25
12.14	1,3,8,9-tetrahydroxy-[1]benzofuro[3,2-c]chromen-6-one (**Demethylwedelolactone**)	C_15_H_8_O_7_	299.02	1.96
*C. hereroense*				
4.68	(10S,11R,12R,13R,15R)-3,4,5,11,12,13,21,22,23,26,27,38,39-tridecahydroxy-9,14,17,29,36-pentaoxaoctacyclo[29.8.0.02,7.010,15.019,24.025,34.028,33.032,37]nonatriaconta-1(39),2,4,6,19,21,23,25,27,31,33,37-dodecaene-8,18,30,35-tetrone (**Punicalin**)	C_34_H_22_O_22_	781.06	3.16
4.99	[(1S,19R,21S,22R,23R)-6,7,8,11,12,13,22,23-octahydroxy-3,16-dioxo-2,17,20-trioxatetracyclo[17.3.1.04,9.010,15]tricosa-4,6,8,10,12,14-hexaen-21-yl] 3,4,5-trihydroxybenzoat (**Corilagin**)	C_27_H_22_O_18_	633.07	1.56
5.20	(2R,3R,4S)-2-(3,4-dihydroxyphenyl)-4-[(2R,3R)-2-(3,4-dihydroxyphenyl)-3,5,7-trihydroxy-3,4-dihydro-2H-chromen-8-yl]-8-[(2R,3R,4R)-2-(3,4-dihydroxyphenyl)-3,5,7-trihydroxy-3,4-dihydro-2H-chromen-4-yl]-3,4-dihydro-2H-chromene-3,5,7-triol (**Procyanidin C1**)	C_45_H_38_O_18_	865.20	2.88
6.14	3,5-dimethoxy-4-[(2S,3R,4S,5S,6R)-3,4,5-trihydroxy-6-(hydroxymethyl)oxan-2-yl]oxybenzoic acid (**Glucosyringic acid**)	C_15_H_20_O_10_	359.10	3.43
6.42	2R,3S)-2-(3,4-dihydroxyphenyl)-8-[(2R,3R,4R)-2-(3,4-dihydroxyphenyl)-3,5,7-trihydroxy-3,4-dihydro-2H-chromen-4-yl]-3,4-dihydro-2H-chromene-3,5,7-triol (**Procyanidin B1**)	C_30_H_26_O_12_	577.14	−4.74
6.64	(1R,3R,4S,5R)-1,3,4-trihydroxy-5-[(E)-3-(4-hydroxyphenyl)prop-2-enoyl]oxycyclohexane-1-carboxylic acid (**3-p-Coumaroylquinic acid**)	C_16_H_18_O_8_	337.09	2.25
6.65	(2R,3R)-2-(3,4-dihydroxyphenyl)-3,4-dihydro-2H-chromene-3,5,7-triol (**Epicatechin**)	C_15_H_14_O_6_	579.15	−2.94
6.81	(2R,3R,4S)-2-(3,4-dihydroxyphenyl)-4-[(2R,3R)-2-(3,4-dihydroxyphenyl)-3,5,7-trihydroxy-3,4-dihydro-2H-chromen-8-yl]-8-[(2R,3R,4R)-2-(3,4-dihydroxyphenyl)-3,5,7-trihydroxy-3,4-dihydro-2H-chromen-4-yl]-3,4-dihydro-2H-chromene-3,5,7-triol (**Procyanidin trimer**)	C_45_H_38_O_18_	865.20	−0.48
7.02	(1S,3R,4R,5R)-3-[(E)-3-(3,4-dihydroxyphenyl)prop-2-enoyl]oxy-1,4,5-trihydroxycyclohexane-1-carboxylic acid (**Chlorogenic acid**)	C_16_H_18_O_9_	359.09	2.37
7.19	(2R,3R)-2-(3,4-dihydroxyphenyl)-8-[(2R,3R,4R)-2-(3,4-dihydroxyphenyl)-3,5,7-trihydroxy-3,4-dihydro-2H-chromen-4-yl]-3,4-dihydro-2H-chromene-3,5,7-triol (**Procyanidin B2**)	C_30_H_26_O_12_	577.14	1.56
8.09	(2R,3S)-2-(3,4-dihydroxyphenyl)-8-[(2R,3S,4S)-2-(3,4-dihydroxyphenyl)-3,5,7-trihydroxy-3,4-dihydro-2H-chromen-4-yl]-3,4-dihydro-2H-chromene-3,5,7-triol (**Procyanidin B3**)	C_30_H_26_O_12_	578.13	−1.03
8.33	(2S,3R,4S)-2-[(2S,3R,4S,5S,6R)-3-[(E)-3-(3,4-dihydroxyphenyl)prop-2-enoyl]oxy-4,5-dihydroxy-6-(hydroxymethyl)oxan-2-yl]oxy-3-ethenyl-4-(2-hydroxyethyl)-3,4-dihydro-2H-pyran-5-carboxylic acid	C_25_H_30_O_13_	537.16	4.61
8.67	2-(3,4-dihydroxyphenyl)-5,7-dihydroxy-8-[(2S,3R,4R,5S,6R)-3,4,5-trihydroxy-6-(hydroxymethyl)oxan-2-yl]chromen-4-one (**Orientin**)	C_21_H_20_O_11_	447.09	2.73
9.01	[(2R,3S,4R,5R,6R)-6-[2-(3,4-dihydroxyphenyl)ethoxy]-4,5-dihydroxy-2-(hydroxymethyl)oxan-3-yl] (E)-3-(3,4-dihydroxyphenyl)prop-2-enoate (**Calceolarioside A**)	C_23_H_26_O_11_	477.14	0.46
9.37	2-(3,4-dihydroxyphenyl)-5,7-dihydroxy-3-[(2S,3R,4S,5R)-3,4,5-trihydroxyoxan-2-yl]oxychromen-4-one (**Reynoutrin**)	C_20_H_18_O_11_	434.3	−7.61
9.42	[(2R,3R,4R,5R,6R)-6-[2-(3,4-dihydroxyphenyl)ethoxy]-5-hydroxy-2-(hydroxymethyl)-4-[(2S,3R,4R,5R,6S)-3,4,5-trihydroxy-6-methyloxan-2-yl]oxyoxan-3-yl] (E)-3-(3,4-dihydroxyphenyl)prop-2-enoate (**Acteoside**)	C_29_H_36_O_15_	623.20	5.08
9.52	[(2R,3S,4S,5R,6S)-6-[5,7-dihydroxy-2-(4-hydroxyphenyl)-4-oxochromen-3-yl]oxy-3,4,5-trihydroxyoxan-2-yl]methyl 3,4,5-trihydroxybenzoate (**Astragalin 6′′-gallate**)	C_28_H_24_O_15_	599.10	−0.02
9.55	(5,7-dihydroxy-2-(4-hydroxyphenyl)-3-[(2S,4R,5S)-3,4,5-trihydroxy-6-(hydroxymethyl)oxan-2-yl]oxychromen-4-one) (**Kaempferol-3-O-glucoside**)	C_21_H_20_O_11_	447.09	2.6
9.73	5,7-dihydroxy-2-(4-hydroxyphenyl)-3-[(2S,3R,4S,5S,6R)-3,4,5-trihydroxy-6-[[(2R,3R,4R,5R,6S)-3,4,5-trihydroxy-6-methyloxan-2-yl]oxymethyl]oxan-2-yl]oxychromen-4-one (**nicotiflorin**)	C_27_H_30_O_15_	593.15	0.78
9.99	(2R)-3-(3,4-dihydroxyphenyl)-2-[(E)-3-(3,4-dihydroxyphenyl)prop-2-enoyl]oxypropanoic acid (**rosmarinic acid**)	C_18_H_16_O_8_	359.08	−1.0
9.95	2-(3,4-dihydroxyphenyl)-5,7-dihydroxy-3-[(2S,3R,4R,5R,6S)-3,4,5-trihydroxy-6-methyloxan-2-yl]oxychromen-4-one (**Quercetin-3-O-rhamnoside**)	C_21_H_20_O_11_	447.09	2.66
10.04	(2S)-5-hydroxy-2-(3-hydroxy-4-methoxyphenyl)-7-[(2S,3R,4S,5S,6R)-3,4,5-trihydroxy-6-[[(2R,3R,4R,5R,6S)-3,4,5-trihydroxy-6-methyloxan-2-yl]oxymethyl]oxan-2-yl]oxy-2,3-dihydrochromen-4-one (**Hesperidin**)	C_28_H_34_O_15_	609.18	1.15
10.03	[(1S,2S,3S,4R,5R,6S,7S,9R,10R,12R)-3,4,5,7,12-pentaacetyloxy-6-(acetyloxymethyl)-2-hydroxy-2,10-dimethyl-8-oxo-11-oxatricyclo[7.2.1.01,6]dodecan-10-yl]methyl acetate	C_29_H_38_O_17_	657.21	5.6
10.16	5-hydroxy-2-(3-hydroxy-4-methoxyphenyl)-7-[(2S,3R,4S,5S,6R)-3,4,5-trihydroxy-6-[[(2R,3R,4R,5R,6S)-3,4,5-trihydroxy-6-methyloxan-2-yl]oxymethyl]oxan-2-yl]oxychromen-4-one (**Diosmin**)	C_28_H_32_O_15_	607.17	0.12
10.06	5-[[6-[5,7-dihydroxy-2-(4-hydroxyphenyl)-4-oxochromen-3-yl]oxy-3,4,5-trihydroxyoxan-2-yl]methoxy]-3-hydroxy-3-methyl-5-oxopentanoic acid	C_27_H_28_O_15_	591.14	7.79
10.15	[(2R,3S,4S,5R,6R)-3,4,5-trihydroxy-6-[(2S,3S,4R,5R)-4-hydroxy-3-[(E)-3-(4-hydroxy-3,5-dimethoxyphenyl)prop-2-enoyl]oxy-2,5-bis(hydroxymethyl)oxolan-2-yl]oxyoxan-2-yl]methyl (E)-3-(4-hydroxy-3,5-dimethoxyphenyl)prop-2-enoate (**3′,6-Disinapoylsucrose**)	C_34_H_42_O_19_	753.22	−1.1
10.21	(3R,4R)-4-[(4-hydroxy-3-methoxyphenyl)methyl]-3-[[3-methoxy-4-[(2S,3R,4S,5S,6R)-3,4,5-trihydroxy-6-(hydroxymethyl)oxan-2-yl]oxyphenyl]methyl]oxolan-2-one (**matairesinoside**)	C_26_H_32_O_11_	519.19	2.36
10.35	methyl (4S,5E,6S)-4-[2-[2-(3,4-dihydroxyphenyl)ethoxy]-2-oxoethyl]-5-ethylidene-6-[(2S,3R,4S,5S,6R)-3,4,5-trihydroxy-6-(hydroxymethyl)oxan-2-yl]oxy-4H-pyran-3-carboxylate (**Oleuropein**)	C_25_H_32_O_13_	539.18	−1.01
10.37	5,7-dihydroxy-2-(4-hydroxyphenyl)-3-[(2S,3R,4R,5R,6S)-3,4,5-trihydroxy-6-methyloxan-2-yl]oxychromen-4-one (**Kaempferol-3-O-rhamnoside**)	C_21_H_20_O_10_	431.10	3.97
10.54	1,3,6,7-tetrahydroxyxanthen-9-one (**Norathyriol**)	C_13_H_8_O_6_	259.03	2.0
10.86	[(2S,3R,4S,5S,6R)-3,4,5-trihydroxy-6-(hydroxymethyl)oxan-2-yl] (1R,2R,4aS,6aR,6aS,6bR,8aR,9R,10R,11R,12aR,14bS)-1,10,11-trihydroxy-9-(hydroxymethyl)-1,2,6a,6b,9,12a-hexamethyl-2,3,4,5,6,6a,7,8,8a,10,11,12,13,14b-tetradecahydropicene-4a-carboxylate (**Dotorioside II**)	C_36_H_58_O_11_	711.40	1.99
10.80	(2S,3R,4S,5S,6R)-2-[4-[(3R,3aR,6S,6aR)-3-(3,4-dimethoxyphenyl)-1,3,3a,4,6,6a-hexahydrofuro[3,4-c]furan-6-yl]-2-methoxyphenoxy]-6-(hydroxymethyl)oxane-3,4,5-triol (**Phillyrin**)	C_27_H_34_O_11_	534.6	0.17
10.89	7-[4,5-dihydroxy-6-(hydroxymethyl)-3-(3,4,5-trihydroxy-6-methyloxan-2-yl)oxyoxan-2-yl]oxy-5-hydroxy-2-(4-methoxyphenyl)chromen-4-one (**Acacetin-7-O-neohesperidoside**)	C_28_H_32_O_14_	592.5	3.04
11.08	2-(3,4-dihydroxyphenyl)-5,7-dihydroxy-3-methoxychromen-4-one (**3-O-methylquercetin**)	C_16_H_12_O_7_	315.05	2.40
11.69	5,7-dihydroxy-2-(4-hydroxyphenyl)-3-methoxychromen-4-one (**Isokaempferide**)	C_16_H_12_O_6_	299.06	1.11
11.71	7-hydroxy-5-methoxy-2-phenyl-2,3-dihydrochromen-4-one (**alpinetin**)	C_16_H_14_O_4_	269.08	1.28
11.82	5,7-dihydroxy-2-(4-hydroxy-3-methoxyphenyl)-6-methoxychromen-4-one (**Jaceosidin**)	C_17_H_14_O_7_	329.07	0.51
11.90	[(2S,3R,4S,5S,6R)-3,4,5-trihydroxy-6-(hydroxymethyl)oxan-2-yl] (4aS,6aS,6bR,9R,10R,11R,12aR)-10,11-dihydroxy-9-(hydroxymethyl)-2,2,6a,6b,9,12a-hexamethyl-1,3,4,5,6,6a,7,8,8a,10,11,12,13,14b-tetradecahydropicene-4a-carboxylate	C_36_H_58_O_10_	659.40	1.99
12.37	(1S,4aR,6aR,6aS,6bR,8R,8aR,10R,11R,12aR,14bS)-1,8,10,11-tetrahydroxy-2,2,6a,6b,9,9,12a-heptamethyl-1,3,4,5,6,6a,7,8,8a,10,11,12,13,14b-tetradecahydropicene-4a-carboxylic acid	C_30_H_48_O_6_	504.7	3.12
12.40	3,5-dihydroxy-7-methoxy-2-(4-methoxyphenyl)chromen-4-one (**Kaempferol-7,4′-dimethyl ether**)	C_17_H_14_O_6_	313.07	2.10
12.40	5,7-dihydroxy-2-(4-hydroxy-3,5-dimethoxyphenyl)chromen-4-one (**Tricin**)	C_17_H_14_O_7_	329.07	1.27
12.63	(2-(3,4-dimethoxyphenyl)-5,7-dihydroxy-6-methoxychromen-4-one) (**Eupatilin**)	C_18_H_16_O_7_	343.08	9.84
12.73	(2S)-5,7-dihydroxy-2-phenyl-2,3-dihydrochromen-4-one (**Pinocembrin**)	C_15_H_12_O_4_	255.07	3.32
12.89	3,5,7-trihydroxy-2-(4-methoxyphenyl)chromen-4-one (**Kaempferol-4′-methyl ether**)	C_16_H_12_O_6_	299.06	1.97
13.03	(5S)-5-hydroxy-1-(4-hydroxy-3-methoxyphenyl)decan-3-one (**6-Gingerol**)	C_17_H_26_O_4_	293.18	2.84
13.07	(E)-1-(2,6-dihydroxy-4-methoxyphenyl)-3-phenylprop-2-en-1-one (**Pinostrobin chalcone**)	C_16_H_14_O_4_	269.08	0.08
13.14	5,7-dihydroxy-3-methoxy-2-(4-methoxyphenyl)chromen-4-one (**Ermanin**)	C_17_H_14_O_6_	313.07	1.04
13.70	1-(2,6-dihydroxy-4-methoxyphenyl)-3-phenylpropan-1-one (**2′,6′-dihydroxy-4′-methoxydihydrochalcone**)	C_16_H_16_O_4_	271.10	4.07
*O. europaea* subsp. *africana*				
5.26	2-hydroxy-5-[(2S,3R,4S,5S,6R)-3,4,5-trihydroxy-6-(hydroxymethyl)oxan-2-yl]oxybenzoic acid (**gentisic acid 5-O-beta-glucoside**)	C_13_H_16_O_9_	315.07	−0.50
5.94	(1R,3R,4S,5R)-3-[(E)-3-(3,4-dihydroxyphenyl)prop-2-enoyl]oxy-1,4,5-trihydroxycyclohexane-1-carboxylic acid (**Neochlorogenic acid**)	C_16_H_18_O_9_	353.09	3.36
6.14	3,5-dimethoxy-4-[(2S,3R,4S,5S,6R)-3,4,5-trihydroxy-6-(hydroxymethyl)oxan-2-yl]oxybenzoic acid (**Glucosyringic acid**)	C_15_H_20_O_10_	359.10	3.43
6.46	7-hydroxy-6-[3,4,5-trihydroxy-6-(hydroxymethyl)oxan-2-yl]oxychromen-2-one (**6,7-Dihydroxycoumarin-6-glucoside**)	C_15_H_16_O_9_	339.07	1.73
7.02	(1S,3R,4R,5R)-3-[(E)-3-(3,4-dihydroxyphenyl)prop-2-enoyl]oxy-1,4,5-trihydroxycyclohexane-1-carboxylic acid (**Chlorogenic acid**)	C_16_H_18_O_9_	359.09	2.37
7.17	(2S,3R,4S,5S,6R)-2-[4-[4-[hydroxy-(4-hydroxy-3-methoxyphenyl)methyl]-3-(hydroxymethyl)oxolan-2-yl]-2-methoxyphenoxy]-6-(hydroxymethyl)oxane-3,4,5-triol (**Lanicepside B**)	C_26_H_34_O_12_	537.20	−2.39
7.84	1,3,6,7-tetrahydroxy-2-[(2S,3R,4R,5S,6R)-3,4,5-trihydroxy-6-(hydroxymethyl)oxan-2-yl]xanthen-9-one (**Mangiferin**)	C_19_H_18_O_11_	421.08	3.40
7.92	5,7-dihydroxy-3-(4-hydroxyphenyl)-6,8-bis[3,4,5-trihydroxy-6-(hydroxymethyl)oxan-2-yl]chromen-4-one	C_27_H_30_O_15_	593.15	0.26
7.99	[(2R,3S,4S,5R,6R)-6-[(2S,3S,4S,5R)-3,4-dihydroxy-2,5-bis(hydroxymethyl)oxolan-2-yl]oxy-3,4,5-trihydroxyoxan-2-yl]methyl (E)-3-(4-hydroxy-3,5-dimethoxyphenyl)prop-2-enoate (**Arillatose B**)	C_22_H_30_O_14_	517.16	4.28
8.67	2-(3,4-dihydroxyphenyl)-5,7-dihydroxy-8-[(2S,3R,4R,5S,6R)-3,4,5-trihydroxy-6-(hydroxymethyl)oxan-2-yl]chromen-4-one **(Orientin)**	C_21_H_20_O_11_	447.09	2.73
9.01	[(2R,3S,4R,5R,6R)-6-[2-(3,4-dihydroxyphenyl)ethoxy]-4,5-dihydroxy-2-(hydroxymethyl)oxan-3-yl] (E)-3-(3,4-dihydroxyphenyl)prop-2-enoate **(Calceolarioside A)**	C_23_H_26_O_11_	477.14	0.46
9.01	(2S,3R,4S,5S,6R)-2-[4-[(3S,3aR,6R,6aS)-6-[3,5-dimethoxy-4-[(2S,3R,4S,5S,6R)-3,4,5-trihydroxy-6-(hydroxymethyl)oxan-2-yl]oxyphenyl]-1,3,3a,4,6,6a-hexahydrofuro[3,4-c]furan-3-yl]-2,6-dimethoxyphenoxy]-6-(hydroxymethyl)oxane-3,4,5-triol (**Eleutheroside E**)	C_34_H_46_O_18_	741.26	4.60
9.24	3-[(2S,3R,4S,5S,6R)-4,5-dihydroxy-6-(hydroxymethyl)-3-[(2S,3R,4R,5R,6S)-3,4,5-trihydroxy-6-methyloxan-2-yl]oxyoxan-2-yl]oxy-5,7-dihydroxy-2-(4-hydroxyphenyl)chromen-4-one (**Kaempferol 3-neohesperidoside**)	C_27_H_30_O_15_	629.12	−8.44
9.42	3-[(2S,3R,4R,5R,6S)-4,5-dihydroxy-6-methyl-3-[(2S,3R,4S,5S,6R)-3,4,5-trihydroxy-6-(hydroxymethyl)oxan-2-yl]oxyoxan-2-yl]oxy-5,7-dihydroxy-2-(4-hydroxyphenyl)chromen-4-one (**Kaempferol-3-O-glucosyl(1-2)rhamnoside**)	C_27_H_30_O_15_	593.15	−1.94
9.42	[(2R,3R,4R,5R,6R)-6-[2-(3,4-dihydroxyphenyl)ethoxy]-5-hydroxy-2-(hydroxymethyl)-4-[(2S,3R,4R,5R,6S)-3,4,5-trihydroxy-6-methyloxan-2-yl]oxyoxan-3-yl] (E)-3-(3,4-dihydroxyphenyl)prop-2-enoate (**Acteoside**)	C_29_H_36_O_15_	623.20	5.08
9.55	(5,7-dihydroxy-2-(4-hydroxyphenyl)-3-[(2S,4R,5S)-3,4,5-trihydroxy-6-(hydroxymethyl)oxan-2-yl]oxychromen-4-one) (**Kaempferol-3-O-glucoside**)	C_21_H_20_O_11_	447.09	2.60
9.67	(1R,3R,4S,5R)-3,4-bis[[(E)-3-(3,4-dihydroxyphenyl)prop-2-enoyl]oxy]-1,5-dihydroxycyclohexane-1-carboxylic acid (**Isochlorogenic acid C**)	C_2_5H24O_12_	515.12	0.10
9.73	5,7-dihydroxy-2-(4-hydroxyphenyl)-3-[(2S,3R,4S,5S,6R)-3,4,5-trihydroxy-6-[[(2R,3R,4R,5R,6S)-3,4,5-trihydroxy-6-methyloxan-2-yl]oxymethyl]oxan-2-yl]oxychromen-4-one (**nicotiflorin**)	C_27_H_30_O_15_	593.15	0.78
9.87	2S)-7-[(2S,3R,4S,5S,6R)-4,5-dihydroxy-6-(hydroxymethyl)-3-[(2S,3R,4R,5R,6S)-3,4,5-trihydroxy-6-methyloxan-2-yl]oxyoxan-2-yl]oxy-5-hydroxy-2-(4-hydroxyphenyl)-2,3-dihydrochromen-4-one (**Naringin**)	C_27_H_32_O_14_	579.17	−3.47
9.93	7-[(2S,3R,4S,5S,6R)-4,5-dihydroxy-6-(hydroxymethyl)-3-[(2S,3R,4R,5R,6S)-3,4,5-trihydroxy-6-methyloxan-2-yl]oxyoxan-2-yl]oxy-5-hydroxy-2-(4-hydroxyphenyl)chromen-4-one (**Rhoifolin**)	C_27_H_30_O_14_	577.16	−5.43
9.94	2-(3,4-dihydroxyphenyl)-5,7-dihydroxy-3-[(2S,3R,4R,5R,6S)-3,4,5-trihydroxy-6-methyloxan-2-yl]oxychromen-4-one (**Quercetin-3-O-rhamnoside**)	C_21_H_20_O_11_	895.19	0.74
9.99	(2R)-3-(3,4-dihydroxyphenyl)-2-[(E)-3-(3,4-dihydroxyphenyl)prop-2-enoyl]oxypropanoic acid (**rosmarinic acid**)	C_18_H_16_O_8_	359.08	−1.00
9.98	3,5,7-trihydroxy-2-[3-hydroxy-4-[(2S,3R,4S,5S,6R)-3,4,5-trihydroxy-6-(hydroxymethyl)oxan-2-yl]oxyphenyl]chromen-4-one (**Spiraeoside**)	C_21_H_20_O_12_	463.09	3.18
10.00	4-[5-(4-hydroxy-3-methoxyphenyl)-3,4-dimethylfuran-2-yl]-2-methoxyphenol (**alpha-guaiaconic acid**)	C_20_H_20_O_5_	339.12	1.92
10.04	(2S)-5-hydroxy-2-(3-hydroxy-4-methoxyphenyl)-7-[(2S,3R,4S,5S,6R)-3,4,5-trihydroxy-6-[[(2R,3R,4R,5R,6S)-3,4,5-trihydroxy-6-methyloxan-2-yl]oxymethyl]oxan-2-yl]oxy-2,3-dihydrochromen-4-one (**Hesperidin**)	C_28_H_34_O_15_	609.18	1.15
10.06	5-hydroxy-2-(4-hydroxyphenyl)-7-[(3R,4S,5S,6R)-3,4,5-trihydroxy-6-(hydroxymethyl)oxan-2-yl]oxychromen-4-one (**Apigenin-7-O-glucoside**)	C_21_H_20_O_10_	431.10	−1.45
10.03	[(1S,2S,3S,4R,5R,6S,7S,9R,10R,12R)-3,4,5,7,12-pentaacetyloxy-6-(acetyloxymethyl)-2-hydroxy-2,10-dimethyl-8-oxo-11-oxatricyclo[7.2.1.01,6]dodecan-10-yl]methyl acetate	C_29_H_38_O_17_	657.21	5.6
10.16	5-hydroxy-2-(3-hydroxy-4-methoxyphenyl)-7-[(2S,3R,4S,5S,6R)-3,4,5-trihydroxy-6-[[(2R,3R,4R,5R,6S)-3,4,5-trihydroxy-6-methyloxan-2-yl]oxymethyl]oxan-2-yl]oxychromen-4-one (**Diosmin**)	C_28_H_32_O_15_	607.17	0.12
10.14	(2R,3S,4S,5R,6S)-2-(hydroxymethyl)-6-(2-nitrophenoxy)oxane-3,4,5-triol (**beta-D-Glucopyranoside**)	C_12_H_15_O_8_	301.25	−2.39
10.15	[(2R,3S,4S,5R,6R)-3,4,5-trihydroxy-6-[(2S,3S,4R,5R)-4-hydroxy-3-[(E)-3-(4-hydroxy-3,5-dimethoxyphenyl)prop-2-enoyl]oxy-2,5-bis(hydroxymethyl)oxolan-2-yl]oxyoxan-2-yl]methyl (E)-3-(4-hydroxy-3,5-dimethoxyphenyl)prop-2-enoate (**3′,6-Disinapoylsucrose**)	C_34_H_42_O_19_	753.22	−1.1
10.21	(3R,4R)-4-[(4-hydroxy-3-methoxyphenyl)methyl]-3-[[3-methoxy-4-[(2S,3R,4S,5S,6R)-3,4,5-trihydroxy-6-(hydroxymethyl)oxan-2-yl]oxyphenyl]methyl]oxolan-2-one (**matairesinoside**)	C_26_H_32_O_11_	519.19	2.36
10.27	5-hydroxy-2-(4-hydroxy-3-methoxyphenyl)-7-[(2S,3R,4S,5S,6R)-3,4,5-trihydroxy-6-(hydroxymethyl)oxan-2-yl]oxychromen-4-one (**7-Glu Chrysoeriol**)	C_22_H_22_O_11_	461.11	0.85
10.35	methyl (4S,5E,6S)-4-[2-[2-(3,4-dihydroxyphenyl)ethoxy]-2-oxoethyl]-5-ethylidene-6-[(2S,3R,4S,5S,6R)-3,4,5-trihydroxy-6-(hydroxymethyl)oxan-2-yl]oxy-4H-pyran-3-carboxylate (**Oleuropein**)	C_25_H_32_O_13_	539.18	−1.01
10.37	5,7-dihydroxy-2-(4-hydroxyphenyl)-3-[(2S,3R,4R,5R,6S)-3,4,5-trihydroxy-6-methyloxan-2-yl]oxychromen-4-one (**Kaempferol-3-O-rhamnoside**)	C_21_H_20_O_10_	431.10	3.97
10.58	3-[(2S,3R,4S,5S,6R)-4,5-dihydroxy-6-(hydroxymethyl)-3-[(2S,3R,4S,5R)-3,4,5-trihydroxyoxan-2-yl]oxyoxan-2-yl]oxy-2-(3,4-dihydroxyphenyl)-5-hydroxy-7-methoxychromen-4-one	C_27_H_30_O_16_	609.14	−2.46
10.67	2-(3,4-dihydroxyphenyl)-5,7-dihydroxy-6-methoxychromen-4-one (**Nepetin**)	C_16_H_12_O_7_	315.05	0.48
10.80	(2S,3R,4S,5S,6R)-2-[4-[(3R,3aR,6S,6aR)-3-(3,4-dimethoxyphenyl)-1,3,3a,4,6,6a-hexahydrofuro[3,4-c]furan-6-yl]-2-methoxyphenoxy]-6-(hydroxymethyl)oxane-3,4,5-triol (**Phillyrin**)	C_27_H_34_O_11_	534.6	0.17
10.86	(2R,3R,4S,5S,6R)-6-(hydroxymethyl)oxane-2,3,4,5-tetrol (**beta-D-Glucopyranose**)	C_6_H_12_O_6_	180.16	
11.69	5,7-dihydroxy-2-(4-hydroxyphenyl)-3-methoxychromen-4-one (**Isokaempferide**)	C_16_H_12_O_6_	299.06	1.11
11.71	7-hydroxy-5-methoxy-2-phenyl-2,3-dihydrochromen-4-one (**alpinetin**)	C_16_H_14_O_4_	269.08	1.28
11.82	5,7-dihydroxy-2-(4-hydroxy-3-methoxyphenyl)-6-methoxychromen-4-one (**Jaceosidin**)	C_17_H_14_O_7_	329.07	0.51
11.90	[(2S,3R,4S,5S,6R)-3,4,5-trihydroxy-6-(hydroxymethyl)oxan-2-yl] (4aS,6aS,6bR,9R,10R,11R,12aR)-10,11-dihydroxy-9-(hydroxymethyl)-2,2,6a,6b,9,12a-hexamethyl-1,3,4,5,6,6a,7,8,8a,10,11,12,13,14b-tetradecahydropicene-4a-carboxylate	C_36_H_58_O_10_	650.80	1.99
12.14	1,3,8,9-tetrahydroxy-[1]benzofuro[3,2-c]chromen-6-one (**Demethylwedelolactone**)	C_15_H_8_O_7_	299.02	1.96
12.40	5,7-dihydroxy-6-methoxy-2-(4-methoxyphenyl)chromen-4-one (**Pectolinarigenin**)	C_17_H_14_O_6_	313.07	2.10
12.63	(2-(3,4-dimethoxyphenyl)-5,7-dihydroxy-6-methoxychromen-4-one) (**Eupatilin**)	C_18_H_16_O_7_	343.08	9.84
12.73	(2S)-5,7-dihydroxy-2-phenyl-2,3-dihydrochromen-4-one (**Pinocembrin**)	C_15_H_12_O_4_	255.07	3.32
13.03	(5S)-5-hydroxy-1-(4-hydroxy-3-methoxyphenyl)decan-3-one (**6-Gingerol**)	C_17_H_26_O_4_	293.18	2.84
13.70	1-(2,6-dihydroxy-4-methoxyphenyl)-3-phenylpropan-1-one (**2′,6′-dihydroxy-4′-methoxydihydrochalcone**)	C_16_H_16_O_4_	271.10	1.19

**Table 2:** Quantifications of the total content of phenolic, tannin, flavonoid, and antioxidant activities of the plant extracts.

Plant Species	Total Phenolic Content (mg GAE/g Extract)	Total Tannin Content (mg GAE/g Extract)	Total Flavonoid Content (mg QE/g Extract)	DPPH Radical Scavenging Activity IC_50_ (µg/mL)	Ferric Reducing Power
*B. saligna*	20.18 ± 0.97 ^a^	287.18 ± 0.19 ^c^	16.48 ± 0.05 ^c^	17.66 ± 5.39 ^a,b^	399.1 ± 3.71 ^b^
*C. hereroense*	21.91 ± 0.75 ^a,b^	245.45 ± 0.36 ^b^	1.79 ± 0.13 ^a^	32.16 ± 6.49 ^b^	2238 ± 0.97 ^c^
*O. europaea* subsp. *africana*	27.18 ± 0.77 ^b^	96.84 ± 0.24 ^a^	8.94 ± 0.45 ^b^	57.84 ± 4.88 ^c^	13.934 ± 1.43 ^d^
Ascorbic acid				14.14 ± 1.69 ^a^	93.76 ± 7.57 ^a^

All values are represented as mean ± standard deviation (SD). mg GAE/g: milligrams of gallic acid equivalents per gram; mg QE/g: milligrams of quercetin equivalents per gram. Different superscripts in the values denote significant differences whereby *p* < 0.05 denotes statistical significance for the means in each column. Similar superscripts mean that there was no statistically significant difference (*p* > 0.05).

**Table 3:** Antimycobacterial activity and percentage sliding motility inhibition of stem acetone extracts against *M. smegmatis*.

Antimycobacterial Activity		Percentage Inhibition of Sliding Motility	
Plant name	MIC (mg/mL)	½ MIC	MIC
*B. saligna*	0.31	−10 ± 1.0	10 ± 1.0
*C. hereroense*	0.16	15 ± 1.0	30 ± 1.0
*O. europaea* subsp. *africana*	0.63	−5 ± 1.0	0 ± 1.0
Rifampicin	0.08	75 ± 0.00	100 ± 0.0

(−): negative values indicate enhancement of sliding motility. All values are presented as mean ± standard deviation (SD). MIC: minimum inhibitory concentration.

## Data Availability

The original contributions presented in this study are included in the article. Further inquiries can be directed to the corresponding author.

## Abbreviations

The following abbreviations are used in this manuscript:

TB	Tuberculosis
DPPH	2,2-Diphenyl-1-picrylhydrazy
GPL	Glycopeptidolipids
EPS	Extracellular polymeric substances
MIC	Minimum inhibitory concentration
